# Identification of antigenic epitopes recognized by tumor infiltrating lymphocytes in high grade serous ovarian cancer by multi-omics profiling of the auto-antigen repertoire

**DOI:** 10.1007/s00262-023-03413-7

**Published:** 2023-03-21

**Authors:** Douglas G. Millar, S. Y. Cindy Yang, Azin Sayad, Qingchuan Zhao, Linh T. Nguyen, Kathrin Warner, Ami G. Sangster, Munehide Nakatsugawa, Kenji Murata, Ben X. Wang, Patricia Shaw, Blaise Clarke, Marcus Q. Bernardini, Trevor Pugh, Pierre Thibault, Naoto Hirano, Claude Perreault, Pamela S. Ohashi

**Affiliations:** 1 Tumor Immunotherapy Program, Princess Margaret Cancer Centre, 610 University Avenue, Toronto, ON M5G 2M9 Canada; 2 Institute for Research in Immunology and Cancer, Université de Montréal, Montréal, Québec Canada; 3 Division of Gynecologic Oncology, Cancer Clinical Research Unit (CCRU), Princess Margaret Cancer Centre, Toronto, ON Canada; 4 Department of Immunology, University of Toronto, Toronto, ON Canada

**Keywords:** Auto-antibodies, Seromics, Transcriptomics, Epitope prediction, Tumor-associated antigens, Cancer vaccine

## Abstract

Immunotherapeutic strategies aimed at enhancing tumor cell killing by tumor-specific T cells hold great potential for reducing tumor burden and prolonging survival of cancer patients. Although many potential tumor antigens have been described, identifying relevant targets when designing anti-cancer vaccines or targeted cell therapies remains a challenge. To identify novel, potentially immunogenic candidate tumor antigens, we performed integrated tumor transcriptomic, seromic, and proteomic analyses of high grade serous ovarian cancer (HGSC) patient tumor samples. We identified tumor neo-antigens and over-expressed antigens using whole exome and RNA sequencing and examined these in relation to patient-matched auto-antibody repertoires. Focusing on MHC class I epitopes recognized by CD8^+^ T cells, HLA-binding epitopes were identified or predicted from the highly expressed, mutated, or auto-antibody target antigen, or MHC-associated peptides (MAPs). Recognition of candidate antigenic peptides was assessed within the tumor-infiltrating T lymphocyte (TIL) population expanded from each patient. Known tumor-associated antigens (TAA) and cancer/testis antigens (CTA) were commonly found in the auto-antibody and MAP repertoires and CD8^+^ TILs recognizing epitopes from these antigens were detected, although neither expression level nor the presence of auto-antibodies correlated with TIL recognition. Auto-antibodies against tumor-mutated antigens were found in most patients, however, no TIL recognition of the highest predicted affinity neo-epitopes was detected. Using high expression level, auto-antibody recognition, and epitope prediction algorithms, we identified epitopes in 5 novel antigens (MOB1A, SOCS3, TUBB, PRKAR1A, CCDC6) recognized by HGSC patient TILs. Furthermore, selection of epitopes from the MAP repertoire identified 5 additional targets commonly recognized by multiple patient TILs. We find that the repertoire of TIL specificities includes recognition of highly expressed and immunogenic self-antigens that are processed and presented by tumors. These results indicate an ongoing autoimmune response against a range of self-antigens targeted by HGSC TILs.

**Supplementary Information:**

The online version contains supplementary material available at 10.1007/s00262-023-03413-7.

## Introduction

Therapeutic vaccination against tumor-expressed antigens can promote tumor-specific T cell-mediated killing of cancer cells and represents a promising form of immunotherapy for cancer. Tumor target antigens fall broadly into two classes: tumor-*associated* antigens (TAAs), including tissue differentiation antigens, tumor over-expressed antigens, and cancer/testis antigens (CTA); and tumor-*specific* antigens (TSA) including mutated neo-antigens and aberrantly expressed translation products from non-canonical reading frames [1–3]. Neo-antigens arise from somatic mutations in tumor-expressed protein-coding regions and can include non-synonymous single nucleotide variants (SNV) leading to amino acid substitutions or insertions/deletions generating altered reading frames. While “driver” mutations in oncogenes and tumor suppressors, such as IDH1, KRAS and TP53, can give rise to antigenic neo-epitopes [4–6], “passenger” mutations resulting from dysregulated tumor DNA replication/repair processes, and that are elevated in environmentally exposed tissue sites (e.g. skin, lung, colon), are thought to provide the greatest source of neo-epitopes [7].

A large number of TAAs that can be recognized by cytotoxic T lymphocytes (CTL) and mediate tumor rejection have been described and a number of clinical trials using vaccination against TAAs to boost therapeutic anti-tumor immunity are ongoing [8]. Limitations and concerns when using shared, non-tumor-specific TAA vaccination included pre-existing tolerance against the endogenous antigen(s) and the possibility of on-target/off-tumor tissue destruction. Neo-antigen TSAs, on the other hand, represent new “foreign” antigenic elements not previously encountered by the immune system, and thus may be more immunogenic. Mutated sequences identified in melanoma patient tumors were found to be recognized by expanded TILs [9, 10] and subsequently, neo-antigen-specific TILs were shown to be therapeutic in melanoma [11], cholangiocarcinoma [12], and breast cancer [13]. Neo-epitopes were demonstrated to be immunogenic when administered as peptide, peptide-pulsed dendritic cells, or RNA-encoded vaccines [14–17]. There is currently great interest in determining which mutated neo-antigens or other tumors antigens are immunogenic in cancer patients, in order to design personalized cancer vaccines or targeted cell-based immunotherapies.

High grade serous ovarian cancer (HGSC) remains one of the most common and lethal subtypes of ovarian cancer affecting women [18]. Despite the ability to treat some HGSC patients with effective chemotherapies including taxol, platinum, and PARP-inhibitors, tumor growth often progresses or recurs, and 5-year survival prognosis remains low [18]. Additionally, HGSC patients respond poorly to immunotherapies [19, 20]. HGSC is characterized by homologous repair defects and frequent mutations in TP53 and BRCA1/2, with large-scale and focal chromosomal copy number alteration but relatively few SNV substitutions [21]. Despite this paucity of potential neo-antigens in HGSC, several groups have reported detection of neo-epitope-specific T cells within TIL populations from epithelial ovarian cancer patients [6, 22–25], indicating that targeting neo-antigen with personalized vaccines or cell therapies may be feasible therapeutic approaches for these patients.

Historically, several TAAs were discovered using serological identification of antigens by recombinant expression cloning or “SEREX” [26]. The co-occurrence of antigen recognition by serum IgG antibodies with the presence of CD4^+^ and CD8^+^ T cell specific for linear epitopes in the same antigen is supported by the requirement for CD4^+^ T cell help for Ig class switching, and for enhancing CD8^+^ T cell memory cell development [27], and linked immunity to the same antigen in the humoral and cellular compartments has frequently been reported [28–30].

Advances in the accuracy of prediction algorithms has allowed candidate epitopes from target proteins or mutated genes to be inferred from gene sequence alone [31, 32]. Despite these advances, selection of tumor antigens that will be recognized by individual patient T cell repertoires and discovery of novel patient-specific epitopes for use in personalized vaccines remains challenging, with no clear optimal method.

In this study, we have used a combination of tumor whole exome sequencing, transcriptomics (RNA-seq), seromics (auto-antibody identification using protein arrays), and identification of MHC-associated peptides (MAPs) by mass-spectrometry to perform an integrated analysis of the antigen specificities of CD8^+^ TILs from HGSC patients. Novel antigenic targets, defined by auto-antibody recognition, high tumor gene expression, and high affinity epitope prediction, were found to be recognized by CD8^+^ T cells from HGSC patient tumors. In addition, the TIL repertoire contained reactivity against previously described T cell epitopes, particularly those that were identified by mass spectrometry as MAPs eluted from HGSC tumors. The suitability of these target antigens as vaccine components is discussed.

## Results

### Auto-antibodies in HGSC patients recognize a variety of tumor-expressed antigens

**Fig. 1:**
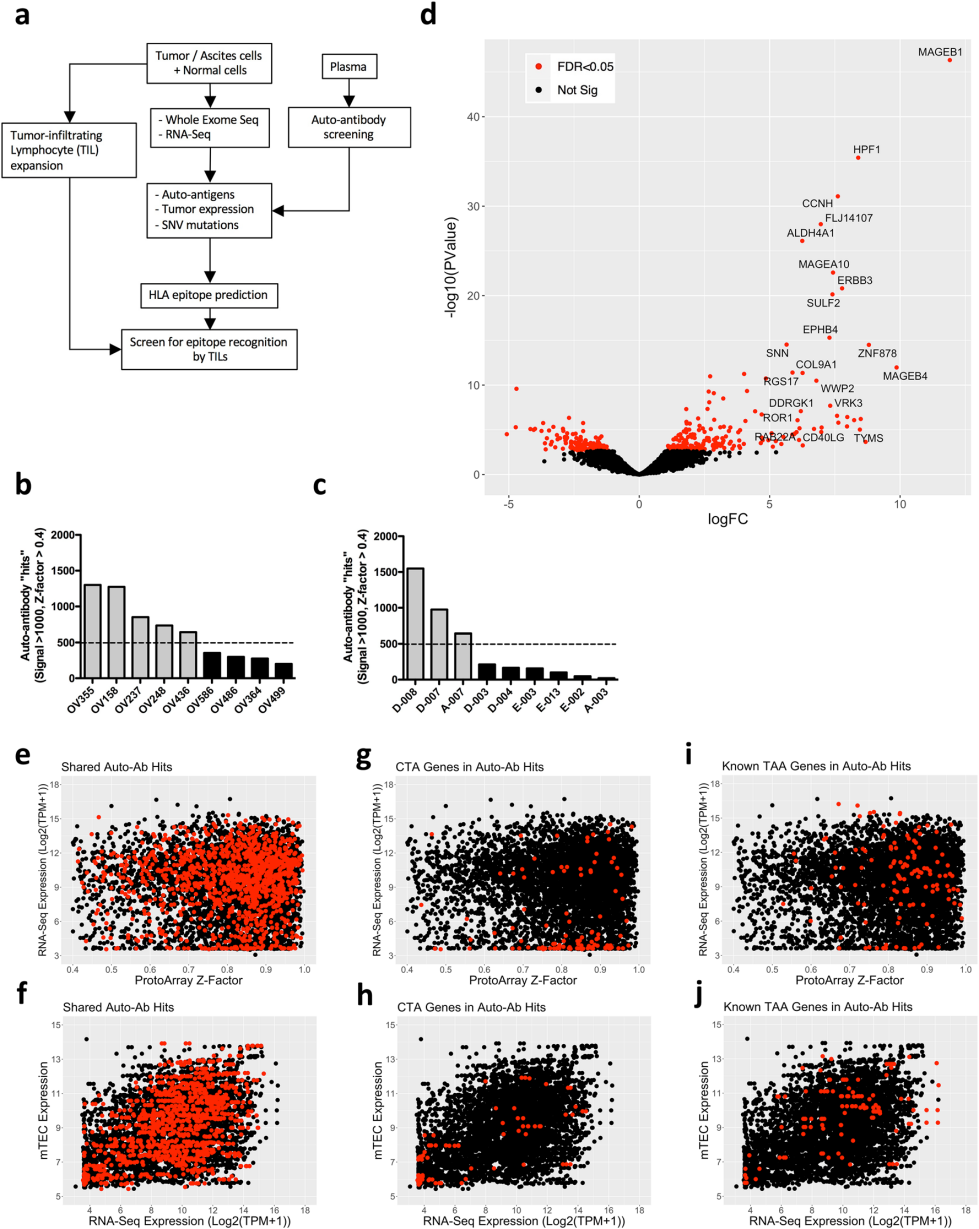
The auto-antibody repertoire of HGSC patients targets highly expressed tumor genes and known TAA. **a** Schematic workflow of multi-omics approach used in the study. **b, c** Number of significant auto-antibody “hits” from HGSC (**b**) and non-HGSC (**c**) patients identified by ProtoArray. Patients with high auto-antibody counts (> 500 target hits, dotted line), and low counts (< 500 targets) were present in both groups. **d** Differential gene expression (edgeR) analysis of the HGSC auto-antibody repertoire compared to non-HGSC patients. Normalized ProtoArray signals were used for 9 samples in each group. **e** Tumor RNA-seq gene expression (TPM) and auto-antibody target gene ProtoArray *Z*-Factor were used to plot all significant hits (*Z*-Factor > 0.4) from all HGSC patients (black circles), and targets differentially enriched in the HGSC group or shared between at least 4 of 9 patient samples are highlighted (red circles). **f** Auto-antibody target gene expression (RNA-seq TPM) versus thymic mTEC expression (from Ref. [35]) was similarly plotted for all HGSC patient auto-antibody hits (black circles), and shared targets (red circles). **g-h** CT antigens (red circles) were plotted by tumor expression versus ProtoArray *Z*-Factor (**g**) or thymic mTEC expression versus tumor expression (**h**) and compared with total auto-antibodies hits from all samples (black circles). **i–j** Tumor and thymic mTEC expression level of cancer antigen genes with reported T cell epitopes from the TANTIGEN database (red circles), plotted over total auto-antibody hits (black circles)

To investigate the potential repertoire of immunogenic TAAs recognized by HGSC patient TILs, we first took an integrated approach combining genomic, transcriptomic and seromic analysis of patient samples, as illustrated in Fig. 1a. We selected nine HGSC patients (Supplementary Table S1) expressing HLA-A*02:01, with pre-surgery plasma samples and sufficient solid tumor or ascites cells for both TIL expansion and DNA/RNA extraction for detailed analysis of tumor antigen/neo-antigen expression and recognition. Plasma samples were used to probe high density human protein arrays (ProtoArray 5.1, Invitrogen) to identify the HGSC-associated IgG auto-antibody repertoire. For comparison, the auto-antibody repertoires from nine patients with other cancer types were also determined (Supplementary Table S1). HGSC patients (Fig. 1b) had between 200 and 1301 (mean = 659) auto-antibody “hits” (i.e. with array signal > 1000 units and *Z*-factor > 0.4), and mixed non-HGSC patients had between 21 and 1549 (mean = 430) hits (Fig. 1c, and Supplementary Table S1).

Differential gene expression analysis identified 199 significantly increased auto-antibody signals in plasma from HGSC compared to non-HGSC patients (Fig. 1d). We additionally identified 339 auto-antibody target hits that were shared between at least 4 out of 9 HGSC patients. Only 14 of the shared targets were among the 199 HGSC enriched auto-antibody signals. The total repertoire of 524 genes shared by HGSC patients or enriched compared to non-HGSC patients was compared with previously published ProtoArray data for breast cancer (BC) patients and healthy female donors [33] and ovarian cancer patients [34]. Surprisingly, only 15 of the targets overlapped with the 202 ovarian cancer-specific targets identified in a previous study which used an earlier version of ProtoArrays [34]. To investigate whether the HGSC auto-antibody repertoire was targeting tissue or pathway-specific gene subsets, we assessed enrichment of gene families in the shared HGSC versus healthy donor (HD) or BC repertoires using the molecular signatures database from the Broad Institute (https://www.gsea-msigdb.org/gsea/msigdb/) (Supplementary Fig. S1a, b). Interestingly, HGSC shared auto-antibody targets included 10 genes belonging to the cytokines and growth factors family not seen in the HD or BC shared repertoires (Supplementary Fig. S1b, c). A detailed comparison of auto-antibodies shared between the HD, BC, and HGSC repertoires revealed that most auto-antibody targets were unique to each clinical group, while HGSC and BC showed slightly more overlapping targets (165/524 = 31.5%) than in the HGSC and HD repertoires (148/524 = 28.2%) (Supplementary Fig. S1d). These observations suggest that the HGSC auto-antibody repertoire may be directed largely against personalized, “private” tumor epitopes and against tumor antigens shared with BC patients, in addition to some “public” auto-antigens found in the normal repertoire.

We performed bulk RNA-seq on CD45-negative cells sorted from the HGSC patients’ tumor samples. Shared auto-antibody targets were abundant in highly expressed tumor genes, although genes at the lowest detectable expression levels were also represented, as visualized in plots of ProtoArray *Z*-Factor versus RNA-seq expression level of each target antigen (Fig. 1e). To assess whether the repertoire of auto-antibodies might be influenced by central tolerance of the T cell repertoire, we examined the expression of auto-antibody target genes in a previously published human medullary thymic epithelial cell (mTEC) gene expression dataset [35]. Shared auto-antibody hits were distributed among genes highly expressed in both tumor and mTEC, and also included some genes with low tumor and mTEC expression, but few genes high in mTEC and low in tumor RNA (Fig. 1f). These data indicate that auto-antibody responses are not restricted to over-expressed antigens, nor are they negatively influenced by central tolerance imprinted on T cells by high expression levels in thymic mTEC.

### The HGSC auto-antibody repertoire targets shared tumor-associated antigens

Within the HGSC shared auto-antibody target genes, we noted several CTA genes as well as TAA genes containing known MHC class I epitopes recognized by T cells listed in the TANTIGEN tumor-associated epitope database [36]. The CTA genes in the HGSC patient shared auto-antibody repertoire included XAGE1B, MAGEB1, MAGEB2, CT45A1, and XAGE2 (Fig. 1d). Known TAA genes present in the shared auto-antibody repertoire included ARHGAP17, ANXA2, BIRC7, BCL11A, CDK1, MDM2, and STAT1 (Fig. 1d). We examined the genesets of 201 CTA genes and 398 TANTIGEN genes present in total auto-antibody hits of all nine HGSC patients in tumor expression versus *Z*-Factor plots, and tumor versus thymic mTEC expression plots (Fig. 1g–j). CTA and known T cell epitope genes were identified in the auto-antibody repertoire in both highly expressed and sparingly expressed genes (i.e. most of the CTA), and in genes with mid to low tumor expression (Fig. 1g, i). Most CTA genes exhibited low to moderate thymic mTEC expression, while other known TAA genes were variably expressed in mTECs (Fig. 1h, j). Therefore, auto-antibody responses against known TAA/CTA are also not restricted to over-expressed antigens or constrained by thymic mTEC expression level, since these targets display a range of tumor and mTEC expression levels.

### The auto-antibody repertoire of HGSC patients targets highly tumor-expressed, but not specifically tumor tissue-enriched antigens

To assess whether HGSC patient auto-antibody targets were enriched in HGS tumor over-expressed genes, we examined the TCGA OV PANCAN normalized expression data reflecting ovarian tumor-enriched gene expression [37]. There was an apparent increase of auto-antibodies targeting genes in the TCGA OV PANCAN top 160 genes in the HGSC patients, compared to HD and non-HGSC samples (Supplementary Fig. S2a). However, when we examined the total shared HGSC auto-antibody targets in relation to average TCGA OV PANCAN and RNA-HiSeq gene expression data versus thymic mTEC gene expression, the shared HGSC auto-antibody targets did not appear to be enriched in OV PANCAN gene expression (Supplementary Fig. S2b, PANCAN Expression > 0) but were shifted towards higher average RNA-HiSeq values (Supplementary Fig. S2c, HiSeq Expression > 7). Together, these observations suggest that the auto-antibody repertoire may target highly tumor-expressed, rather than tumor-tissue specific or tissue-enriched antigens.

### Detection of known and novel tumor-associated antigen specificities in HGSC patient TILs

To determine which tumor antigens could be recognized by CD8^+^ T cells, we assessed reactivity of TIL populations against antigenic peptides and tumor cell lines by multiple methods. We selected candidate highly expressed auto-antibody target antigens that contained regions predicted to be high affinity HLA-A*02:01 binding ligands as well as known HLA-A*02:01 epitopes from TANTIGEN and CTA genes represented in the auto-antibody repertoires of the HGSC patients. The genes, epitope sequences, and predicted HLA affinities are shown in Supplementary Table S2.

TILs from each patient were expanded from bulk dissociated tumor fragments or ascites in IL-2, as previously described [38, 39]. TILs were then peptide-expanded with A2^+^ healthy donor monocyte-derived dendritic cells (DCs), pulsed overnight with pooled or individual peptides, for 14 days in low IL-2 high IL-15 (as described in “Materials and methods”), and epitope recognition was determined by peptide re-stimulation, staining with custom peptide-MHC (pMHC) tetramers, or tumor cell re-stimulation on day 14.

**Fig. 2:**
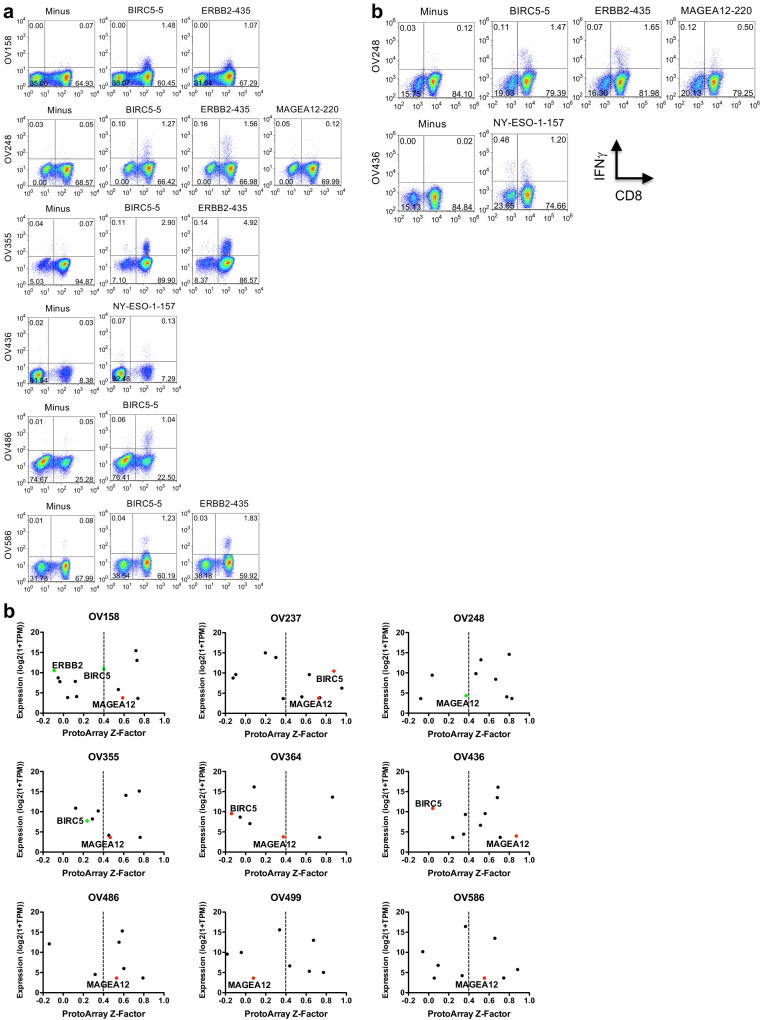
Detection of HGSC patient TILs recognizing known TAA epitopes. **a** Patient TILs were cultured for 14 days with A2^+^ donor DCs pulsed with the 29 peptide pool of known epitopes (*TAA pool*) (see Supplementary Table S2b). On day 14, TILs for each patient were re-stimulated with T2 cells pulsed with individual peptides from the pool (indicated above each *dotplot*), or left unpulsed (*Minus*) for 5 h in the presence of BFA, and the frequency of responding T cells was determined by CD8 surface staining and intracellular IFNγ staining. The percentage of cells in each quadrant is indicated on each plot. Results are representative of 2–3 separate expansion and staining experiments. **b** TILs from patients OV248 and OV436 were expanded with A2^+^ donor DCs pulsed with known TAA epitopes BIRC5-5, ERBB2-435, MAGEA12-220, NY-ESO-1-157, or left unpulsed (*Minus*) for 14 days. On day 14, CD45-negative, epithelial cell lines cultured from the same patients were added back to the peptide-expanded TILs for re-stimulation, for 5 h in the presence of BFA, and the frequency of responding T cells was determined by CD8 surface staining and intracellular IFNγ staining. The percentage of cells in each quadrant is indicated on each plot. Results are representative of 2 separate expansion and re-stimulation experiments. **c** The ProtoArray *Z*-Scores versus individual patient tumor expression (RNA-seq TPM) for each of the genes in the known TAA pool were plotted for each patient. Antigens with detected TIL responses in any patient are labelled with the gene name. Green symbols indicate TIL responses detected in that patients, and red symbols indicate no response detected in that patient

We first examined the response of HGSC TILs to previously characterized TAA epitope peptides (listed in Supplementary Table S2b). In 6 out of 9 patient TILs, we observed IFNγ production in response to individual TAA-peptide re-stimulation (Fig. 2a). Responding cells represented between 0.12 and 4.92% of cultured TILs. Recognition of BIRC5, ERBB2, MAGEA12, and NY-ESO-1 epitopes were detected in different patient TIL samples, with BIRC5 responses being shared between 5 patients, and ERBB2 responses seen in 4 patients. Additionally, T cells specific for individual epitopes were confirmed using custom pMHC tetramers (Supplementary Fig. S3a). To determine if recognized epitopes were endogenously processed and presented by tumor cells, we re-stimulated individual peptide-expanded TILs with matched epithelial cell lines grown from the same patient tumor ascites samples, and observed stimulation of TILs specific for BIRC5, ERBB2, MAGEA12 and NY-ESO-1 epitopes by 2 patient-derived tumors (OV248 and OV436, Fig. 2b). Known TAA epitopes recognized by TILs did not correlate with tumor expression level or auto-antibodies (Fig. 2c). For example, BIRC5 and ERBB2 epitopes were recognized by patients OV158 and OV355 TILs, while these genes did not qualify as “hits” in their auto-antibody repertoires. Similarly, MAGEA12 was recognized by patient OV248, in which expression was very low and auto-antibody *Z*-Factor was less than 0.4, but not in other patients with high MAGEA12 auto-antibody signals (Fig. 2c). Therefore, high antigen expression and/or humoral immunogenicity are not strictly required to have detectable antigen recognition by CD8^+^ TILs.

**Fig. 3:**
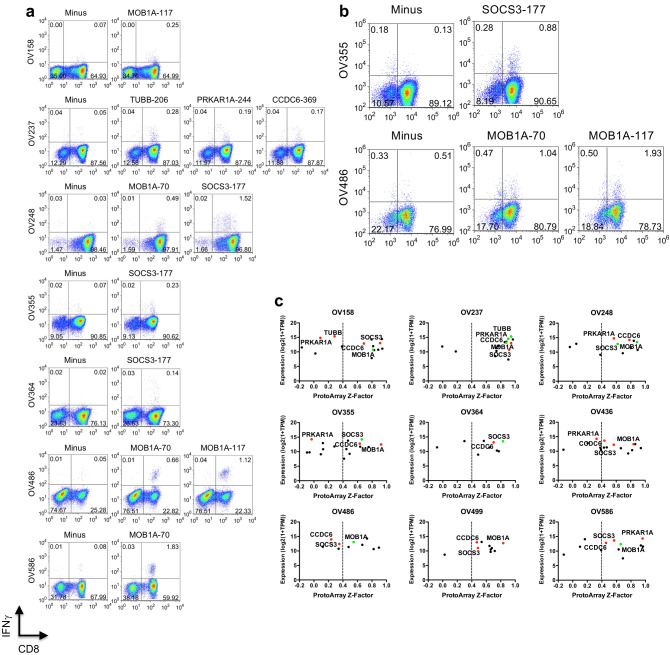
HGSC patient TILs recognize novel antigen epitopes predicted from auto-antibody target genes. **a** Patient TILs were cultured for 14 days with A2^+^ donor DCs pulsed with the 24 peptide pool of predicted epitopes (*AutoAb pool*) selected from the shared and patient-specific auto-antibody repertoires (see Supplementary Table S2c). On day 14, TILs for each patient were re-stimulated with T2 cells pulsed with individual peptides from the pool (indicated above each *dotplot*), or left unpulsed (*Minus*), for 5 h in the presence of BFA, and the frequency of responding T cells was determined by CD8 surface staining and intracellular IFNγ staining. The percentage of cells in each quadrant is indicated on each plot. Results are representative of 2–3 separate expansion and re-stimulation experiments. **b** TILs from patients OV355 and OV486 were expanded with A2^+^ donor DCs pulsed with epitopes SOCS3-177, MOB1A-70 or MOB1A-117, or left unpulsed (*Minus*) for 14 days. On day 14, CD45-negative, epithelial cell lines cultured from the same patients were added back to the peptide-expanded TILs for re-stimulation, for 5 h in the presence of BFA, and the frequency of responding T cells was determined by CD8 surface staining and intracellular IFNγ staining. The percentage of cells in each quadrant is indicated on each plot. Results are representative of 2 separate expansion and re-stimulation experiments. **c** The ProtoArray* Z*-Scores versus individual patient tumor expression (RNA-seq TPM) for each of the genes in the AutoAb pool were plotted for each patient. Antigens with detected TIL responses in any patient are labelled with the gene name. Green symbols indicate TIL responses detected in that patient and red symbols indicate no response detected in that patient

TIL recognition of predicted epitopes from novel auto-antibody target antigens (listed in Supplementary Table S2c) was then examined. We observed auto-antibody target peptide-specific IFNγ production upon re-stimulation with peptide-pulsed target cells in 7 out of 9 samples (Fig. 3a). Responding cells represented between 0.14 and 1.83% of cultured TILs. Recognition of MOB1A, SOCS3, TUBB, PRKAR1A, and CCDC6 epitopes were identified, with responses to MOB1A epitopes shared in 4 patients and responses to SOCS3 seen in 3 patients. CD8^+^ T cells specific for individual epitopes were confirmed using custom pMHC tetramers (Supplementary Fig. S3b). To determine if the recognized epitopes were endogenously processed and presented by tumor cells, we re-stimulated individual peptide-expanded TILs with matched epithelial cell lines grown from the same patient tumor ascites samples and observed stimulation of TILs specific for both MOB1A epitopes and the SOCS3 epitope by 2 patient-derived tumors (OV355 and OV486, Fig. 3b). While the antigens that were recognized by TILs corresponded to highly expressed genes and high auto-antibody *Z*-Factor targets, the presence of auto-antibodies against the target antigens did not guarantee that TIL responses could be detected against the corresponding epitopes. In particular, there were patients with high anti-MOB1A and anti-SOCS3 antibodies who did not show an IFNγ response to the individual peptides and in whom no tetramer-positive TILs were detected (Fig. 3c). However, since we only examined HLA-A2-restricted epitopes, it is possible that other TILs recognizing these antigens in the context of other MHC haplotypes could be present.

### Lack of neo-epitope recognition in HGSC patient TILs

**Fig. 4:**
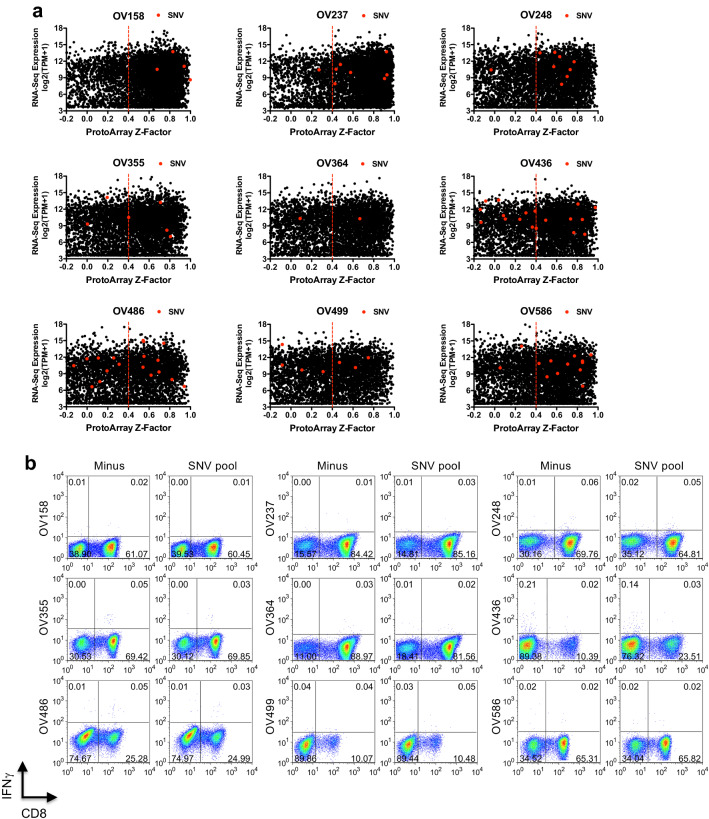
Humoral but not cellular immunity detected against tumor mutated antigens. **a** Total auto-antibody target *Z*-Factors versus tumor expression (RNA-seq TPM) were plotted (black circles) for each patient (indicated above each plot). Expressed genes with single nucleotide variants, identified from whole exome and RNA-seq of each patient, are highlighted (SNV, red circles). The vertical dotted red line indicates the cut-off for significant “hit” (*Z*-Factor > 0.4). **b** The top candidate HLA-A*02:01 epitope peptides from each patient were predicted (see Supplementary Table S2d) and pooled synthetic peptides (*SNV pool*) were used to pulse A2^+^ healthy donor DCs and expand 9 patient TILs for 14 days. On day 14, expanded TILs for each patient were re-stimulated with T2 cells pulsed with the same pool, or left unpulsed (*Minus*) for 5 h in the presence of BFA, and the frequency of responding T cells was determined by CD8 surface staining and intracellular IFNγ staining. The percentage of cells in each quadrant is indicated on each plot. Results are representative of 2–3 separate expansion and re-stimulation experiments

Using whole exome sequencing of tumor/normal pairs and RNA-seq data, we identified the expressed somatic single nucleotide variants (SNV), encoding potential neo-antigens, in each patient. The number of expressed SNVs was between 10 and 67 (Table 1), with mutations in TP53 frequently detected (Supplementary Table S3). We then examined the humoral immunogenicity of the antigens harboring SNV mutations using ProtoArrays, and the potential generation of MHC class I neo-epitopes. Of the genes included on the ProtoArray, many of the mutated genes in each HGSC patient were found to be in the significant auto-antibody hits repertoire (*Z*-Factor > 0.4), whether they were expressed at high or low levels (Fig. 4a). The mutations did not appear to cluster in genes with a low or high thymic mTEC expression level (Supplementary Fig. S4a) or in genes enriched for OV PANCAN expression (Supplementary Fig. S4b) but were mostly in genes with high expression in TCGA OV HiSeq data (Supplementary Fig. S4c). These observations suggest that passenger mutations can occur in highly tumor expressed genes and this may increase their humoral immunogenicity.

**Table 1:** Neo-epitope prediction from 9 HGSC patients

Patient ID	Somatic SNV	HLA-A		HLA-B		HLA-C		A*02:01 Predicted neo-epitopes
OV158	11	A*02:01	A*31:01	B*15:01	B*18:01	C*03:03	C*07:01	2
OV237	30	A*02:01	–	B*40:01	B*44:03	C*03:04	C*16:01	6
OV248	32	A*02:01	–	B*13:01	B*44:05	C*03:04	–	3
OV355	13	A*02:01	A*23:01	B*14:02	B*51:01	C*02:02	C*08:02	2
OV364	10	A*02:01	–	B*15:30	B*18:01	C*03:04	C*07:01	3
OV436	48	A*02:01	A*26:01	B*18:01	B*51:01	C*07:01	C*15:02	6
OV486	67	A*02:01	A*29:02	B*08:01	B*44:03	C*07:01	C*16:01	9
OV499	38	A*02:01	A*30:04	B*44:02	B*49:01	C*07:01	C*15:02	8
OV586	54	A*02:01	A*31:01	B*39:01	B*46:01	C*01:02	–	9

For each patient, total expressed somatic single nucleotide variants (SNV) determined from WES and RNA-seq are shown. HLA typing was deduced by Seq2HLA software and confirmed by PCR. The number of predicted epitopes were determined based on mutated sequences scoring less than 500 nM affinity by NetMHCpan, greater than 0.6 by NetCTLpan combined score, and with greater affinity than the wildtype sequence

For each SNV mutation, we predicted MHC class I epitope processing and HLA binding affinity using the NetCTLpan and NetMHCpan algorithms [31] and selected 2–9 of the highest predicted scoring HLA-A*02:01 epitopes for peptide synthesis and T cell recognition screening of each patient. The peptide epitopes used for TIL expansion and custom pMHC tetramer screening are shown in Supplementary Table S2d.

TILs were cultured for 14 days using A2^+^ healthy donor monocyte-derived DCs pulsed with predicted SNV epitope peptide pools and neo-epitope recognition was assessed by IFNγ-production following peptide re-stimulation. However, as shown in Fig. 4b, none of the 9 patient TILs responded to their neo-epitope pools. To determine whether neo-epitope-specific T cells had expanded but were dysfunctional and unable to produce IFNγ, we stained each peptide-expanded population using custom HLA-A*02:01 tetramers prepared with each SNV neo-epitope peptide. However, no positive staining with any of the 48 top predicted neo-epitopes SNVs was seen in any patient TILs (Supplementary Fig. S5). We were also unable to detect neo-epitope-specific cells using high affinity mutant peptide-HLA-A2 dimer reagents [40] (data not shown). Therefore, using multiple strategies we were unable to detect neo-antigen specific T cells within the expanded CD8^+^ HGSC TIL population.

All HGSC patient TILs examined (9 out of 9) responded with IFNγ production upon re-stimulation with DCs pulsed with peptides of known pathogen-derived epitopes from CMV, EBV, and Flu (CEF pool, listed in Supplementary Table S2a) with patient TILs most frequently recognizing EBV BMLF1 and hCMV pp65 epitopes (Supplemental Fig. S6). Responding cells represented between 0.11 and 22.12% of 14-day peptide-expanded TILs. These results are in agreement with previous observations of viral pathogen epitope recognition by TILs [41–43] and confirm that these “bystander” memory cells frequently infiltrate the ovarian tumor microenvironment and may constitute a high proportion of the TIL reactivity in HGSC patients.

### Recognition of MHC-associated peptides (MAPs) by multiple HGSC patient TILs.

**Fig. 5:**
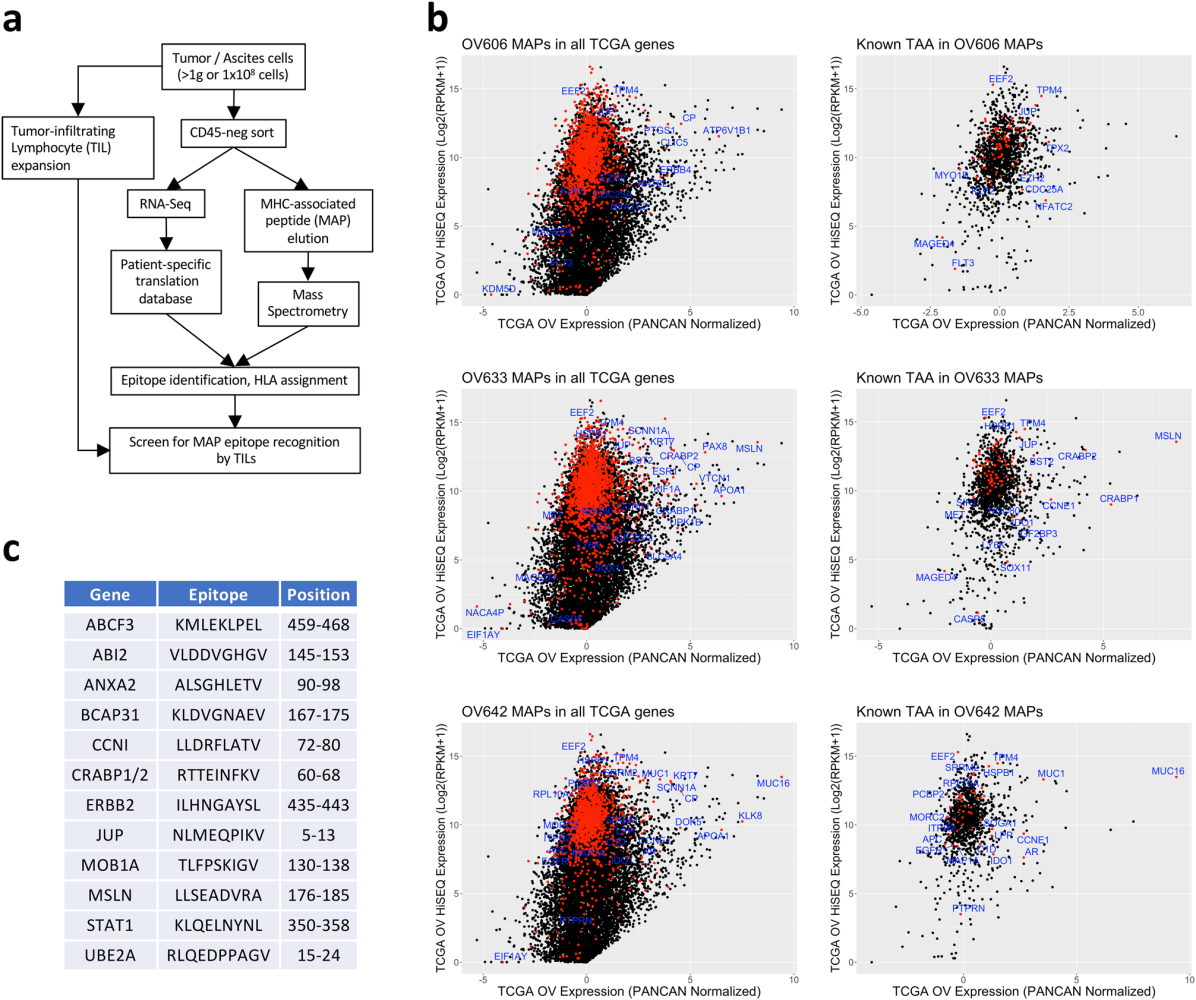
The MAP repertoire of HGSC patients contains epitopes from highly expressed tumor genes and known TAA. **a** Schematic workflow of the mass spectrometry approach to identifying HGSC antigens and screen for recognition by matched patient TILs used in this study. **b** Left side panels: expression values and ovarian cancer enrichment of all MAPs for 3 HGSC patients (OV606, OV633, OV642) are shown (red symbols) relative to all TCGA genes (black symbols) plotted as TCGA OV Illumina HiSEQ RNA-seq expression (log2(RPKM + 1)) versus TCGA OV PANCAN normalized expression. Right side panels show expression levels and ovarian enrichment of known TAA genes (red symbols) relative to total MAPs (black symbols) for each patient sample. **c** HLA-A*02:01 epitopes selected from known TAA and auto-antibody target antigens identified in MAPs from patient sample OV633

To favor selection of antigen targets representing *bona fide* tumor expressed and presented MHC ligands, we screened TILs from patient samples that were subjected to MAP elution and mass spectrometry analyses, reported previously [44]. Patient samples were selected for TIL expansion, tumor cell purification, and MAP elution, as illustrated in the workflow in Fig. 5a. Three patient samples, OV606, OV633 and OV642, yielded 2102, 3029, and 1520 MAPs, respectively (Table 2). We assessed the ovarian cancer enrichment and overall expression level in TCGA OV HiSeq versus PANCAN normalized expression plots and observed generally high expression of transcripts encoding the identified MAPs and several ovary-enriched genes (PANCAN Expresson > 0) including MSLN, MUC16, PAX8, KLK8, and ATP6V1B1 (Fig. 5b left side plots, red symbols). Moreover, the MAP repertoire contained many antigens with known T cell epitopes, contained in the TANTIGEN database [36] and described in previous studies [45], including MSLN, MUC16, CRABP1/2, IDO1, CCNI, JUP, ANXA2, ABI2, BCAP31, CCNI, STAT1, and UBE2A (Fig. 5b, right side panels).

**Table 2:** HLA-types and eluted MAPs from 3 HGSC patients

Patient ID	HLA-A		HLA-B		HLA-C		Eluted MAPs
OV606	A*01:01	A*03:01	B*08:01	B*51:01	C*07:01	C*12:03	2102
OV633	A*02:01	–	B*07:02	B*44:02	C*05:01	C*07:02	3029
OV642	A*11:01	A*24:02	B*18:01	B*52:01	C*07:01	C*12:01	1520

HLA typing of 3 HGSC patients was deduced by Seq2HLA software and confirmed by PCR. The number of eluted MAPs identified for each patient is shown

**Fig. 6:**
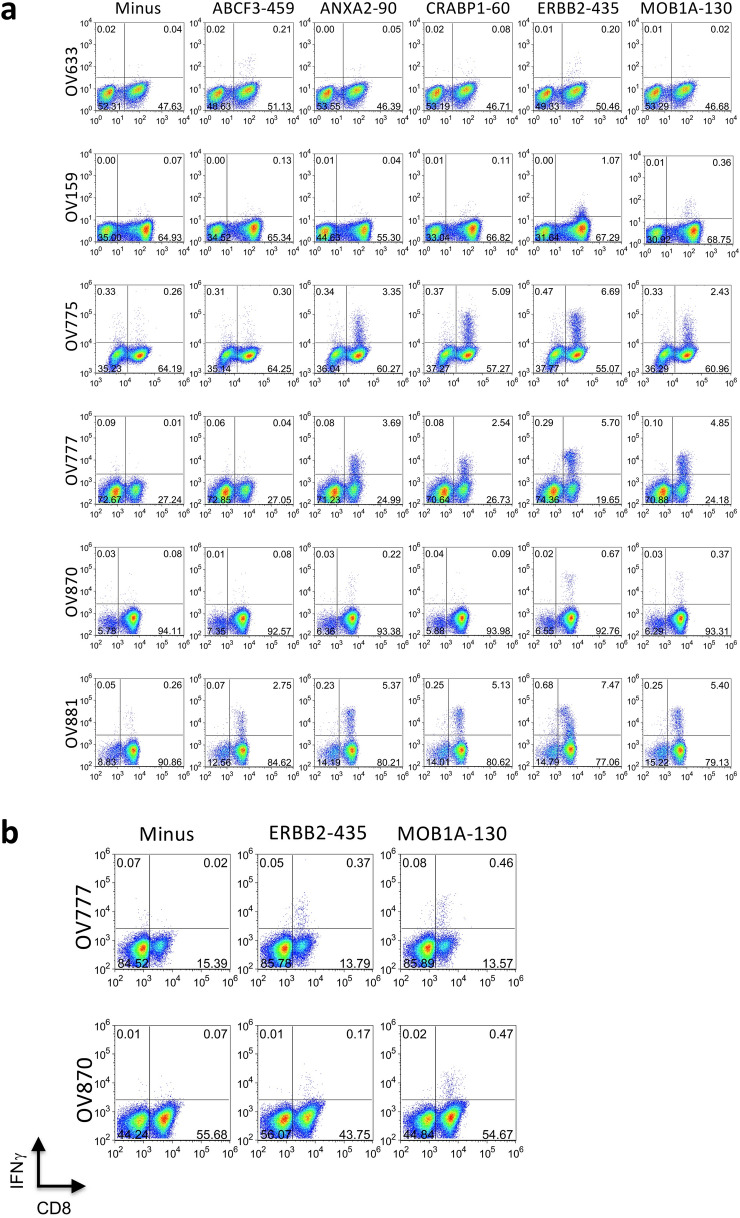
HGSC patient TILs recognize MAPs. **a** TILs from 6 HGSC patients (*rows*) were cultured for 14 days with A2^+^ donor DCs pulsed with the 12 peptide pool of epitopes selected from OV633 eluted MAPs (*MAP pool*) (see Fig. 5c). On day 14, TILs for each patient were re-stimulated with T2 cells pulsed with individual peptides from the pool (indicated in each *column*), or left unpulsed (*Minus*) for 5 h in the presence of BFA, and the frequency of responding T cells was determined by CD8 surface staining and intracellular IFNγ staining. The percentage of cells in each quadrant is indicated on each plot. Results are representative of 2–3 separate expansion and re-stimulation experiments. **b** TILs from patients OV777 and OV870 were expanded with A2^+^ donor DCs pulsed with MAP epitopes ERBB2-435 or MOB1A-130, or left unpulsed (*Minus*) for 14 days. On day 14, CD45-negative, epithelial cell lines cultured from the same patients were added back to the peptide-expanded TILs for re-stimulation for 5 h in the presence of BFA, and the frequency of responding T cells was determined by CD8 surface staining and intracellular IFNγ staining. The percentage of cells in each quadrant is indicated on each plot. Results are representative of 2 separate expansion and re-stimulation experiments

We selected 10 known HLA-A*02:01 epitopes detected in the MAP repertoire of HLA-A2^+^ patient OV633, plus 2 MAPs from proteins with shared auto-antibodies, ABCF3 and MOB1A (Fig. 5c), and screened for their recognition in expanded ascites-derived TIL preparations from 6 additional HGSC patients. Individual peptide re-stimulation demonstrated epitope-specific responses to 5 of the 12 peptides, with responding CD8^+^ T cells representing 0.21–7.47% of total cells (Fig. 6a). To confirm that MAPs were endogenously processed and presented by tumor cells, individual peptide-expanded TILs were re-stimulated with matched epithelial cell lines grown from the same patient tumor ascites samples. Peptide-expanded populations of TILs recognizing either ERBB2 or MOB1A epitopes from two patient samples (OV777 and OV870) responded to matched tumor cells with IFNγ production (Fig. 6b). These results demonstrate that the TIL repertoire in multiple patients may be directed against abundant epitopes known to be displayed by HGSC tumor cells.

To gain insight into whether the identified novel auto-antigens recognized by TILs might be potential HGSC vaccine antigens, we compared their expression across tumor and healthy tissues using a harmonized dataset derived from TCGA tumor RNA-seq and GTEx healthy tissue RNA-seq, using the GEPIA server [46]. While MOB1A and CCDC6 were significantly over-expressed in ovarian cancer samples, expression of these genes, as well as of TUBB, PRKAR1A, and SOCS3 appeared to be expressed at high levels in multiple healthy tissue sites (Supplementary Fig. S7a). Similarly, the MAP-defined antigens ABCF3 and ANXA2 were also highly expressed across healthy tissues, while CRABP2, although significantly over-expressed in ovarian cancer samples, was expressed at similar or greater levels in several healthy sites (Supplementary Fig. S7b). Only the expression profiles of CTA genes from the known TAA (NY-ESO-1 and MAGE-A12) and BIRC5, but not ERBB2, displayed elevated cancer expression with low healthy tissue expression (Supplementary Fig. S7c). Therefore, some of the antigen-specific TILs that are recruited to the TME appear to recognize abundant proteins that are widely expressed in other tissues.

## Discussion

Our study focused on examining MHC class I epitope specificities of CD8^+^ T cells from HGSC patient TILs, in relation to the potentially immunogenic tumor antigens identified from mutated tumor neo-antigens, known over-expressed tumor-antigens, targets of IgG auto-antibody responses, and tumor-presented MAPs. We detected TIL responses against previously reported TAA (BIRC5, ERBB2, MAGEA12, NY-ESO-1), and novel T cell recognition of predicted epitopes derived from auto-antibody targets (MOB1A, SOCS3, TUBB, PRKAR1A, CCDC6). These findings confirm the utility of using seromics as an approach to identifying potential antigen-specificities of TILs. Additionally, we found a prevalence of CD8^+^ T cells specific for pathogen-derived antigens (CMV, EBV, Flu) in all patient TILs. Our data add to the collection of previously described epitopes presented by ovarian tumors and recognized by T cells, and representing potential targets for vaccine, CAR, or TCR-modified cell-based therapies [47].

Surprisingly, no CD8^+^ TILs specific for predicted neo-epitopes in 9 patients were detected in our study. Nelson and co-workers [22], identified T cells recognizing a mutated epitope, HSDL1(L25V), in peripheral blood of a HGSC patient, starting from expanded sub-populations of CD8^+^ T cells. This neo-epitope specificity was then confirmed to be present in ascites fluid tumor-associated lymphocytes. Bobisse et al. identified 10 neo-epitopes recognized by peptide-expanded peripheral blood T cells, one of which (ZCCH) was also found in TILs, and they demonstrated additional neo-antigen T cell detection by expanding peptide-primed T cells from primary tumor tissue [23]. Starting from a pool of 75 candidate predicted neo-epitopes from 20 ovarian cancer patients, Liu et al. identified 5 neo-epitope-specific CD8^+^ T cells in peripheral blood or TILs following expansion by mutant peptide pulsed autologous T cell-depleted PBMC APCs [25]. Neo-epitope-specific T cell identification correlated with higher mutation burden and with an elevated antigen processing machinery gene expression signature [23, 25]. The discordance of neo-epitope recognition in PBMC versus TILs was highlighted by both groups. While these reports suggest that neo-epitope-specific T cells may be present, our findings suggest that neo-antigen-specific TILs in HGSC patients are rare or very low affinity and may be difficult to detect, particularly in the context of only 10–100 mutations per patient. The use of initial neo-epitope priming and expansion protocols using autologous APC, and/or the interrogation of peripheral blood repertoires may be critical for HGSC patient neo-epitope-specific T cell detection.

It is interesting, however, that we observed that a large fraction of the proteins harboring non-synonymous point mutations in each patient were significant targets in the auto-antibody repertoires of those patients (Fig. 4a). This could indicate that the point mutations created new B cell or helper T cell epitopes. This would be consistent with reports that SNV neo-antigen epitopes are frequently recognized by CD4 + T cells, i.e. create new class II epitopes [48, 49]. Indeed, some TP53 mutations are known to raise antibody responses directed against the specific mutated residue [50]. However, a previous study suggested that antibody responses arising following neo-antigen vaccination were directed against the wild-type protein, and not specific for the mutated sequence [30]. It remains to be determined whether auto-antibody responses against neo-antigens are widespread in HGSC or other cancers and if this is due to creation of new CD4^+^ T cell epitopes by tumor mutations.

CTA are frequently re-expressed by tumor cells and due to their low expression in normal tissue and thymus, are thought to be vulnerable to T cell recognition. TCR-engineered cell-based therapies directed against MAGE proteins, NY-ESO-1, and other CTA, as well as anti-tumor vaccines targeting these antigens, have been evaluated as clinical targets in a number of studies [8, 51, 52]. We detected strong auto-antibody responses against CTA in most HGSC patient plasma, and T cell responses against MAGEA12 and NY-ESO-1 in 2 different patients. Surprisingly, detectable T cell recognition of MAGEA12 in patient TILs did not correlate with high levels of auto-antibody, since we did not detect MAGEA12-specific T cells in 4 patients with highly significant anti-MAGEA12 auto-antibodies (Fig. 2a, c). It is possible that MAGEA12 specific T cells in those patients with higher levels of auto-antibodies were functionally impaired in proliferative capacity or cytokine production. Similarly, low IFNγ responses from TILs using peptide that showed high frequency pMHC-tetramer staining would indicate that some auto-antibody target antigen-specific TILs may be dysfunctional.

Our comparison of immunogenic HGSC tumor antigens in the auto-antibody repertoire with a large human thymic mTEC gene expression dataset revealed that auto-antibody target genes can display a range of expression levels in the thymus. This would suggest that, for many tumor antigens, central tolerance of CD4^+^ T cells against thymus-expressed antigens may not necessarily have a negative impact on shaping the anti-tumor B cell or CD8^+^ T cell repertoire. Consequently, given the breadth of tumor-directed anti-self-antibody responses and TIL specificity, T cell response may be directed against more over-expressed self/tumor antigens than previously appreciated.

The most direct method to identify tumor antigen epitopes is by determination of the tumor cell MHC-associated immunopeptidome by mass spectrometry of HLA-eluted peptides. This method can be combined with exome/RNA sequencing to detect presented tissue-specific epitopes, neo-epitopes, and even non-canonical, aberrantly expressed epitopes [44, 45, 53]. We identified several peptides known to be T cell targets in our HGSC MAP data, including ERBB2, MUC16, CRABP1/2, BCAP31 and STAT1, as well epitopes from auto-antibody target antigens, including MOB1A, ABCF3, and ANXA2, and demonstrated recognition of the ERBB2, CRABP1/2, MOB1A, ABCF3 and ANXA2 epitopes by TILs. Interestingly, other peptides from the shared auto-antibody targets with high HGSC tumor expression identified in our screen, including MOB1A, PRKAR1A and CCDC6, have been reported to be present in immunopeptidome datasets (https://hla-ligand-atlas.org). Thus, a considerable fraction of HGSC TIL specificities appears to target highly expressed and immunogenic antigens which are widely expressed and presented by both healthy and tumor cells. This observation is consistent with growing evidence that anti-tumor responses are driven, at least in part, by autoreactive T cells [54]. However, these T cells do not appear to be associated with widespread autoimmunity, despite the ubiquitous expression of their target antigens. One potential explanation for this paradox is that cancer cells are more sensitive than normal cells to effector T cell attack. This concept is supported by the fact that immunotherapy targeting autoantigens can result in melanoma eradication without causing global vitiligo [55, 56].

In summary, we have identified specific recognition of known TAA and novel antigen epitopes in HGSC patient CD8^+^ TILs by combining gene expression profiling, auto-antibody repertoire determination, epitope prediction, and peptide stimulation screening. This provides evidence that HGSC antigens with high tumor expression and humoral immunogenicity may constitute a considerable fraction of targets of CD8^+^ TILs. Directing personalized immunotherapies against these targets may offer additional therapeutic options to enhance tumor cell killing in HGSC patients, however it will be imperative to select T cell targets that have restricted or enriched expression in the tumor to avoid healthy tissue damage.

## Materials and methods

### Patient samples

Tumor samples from standard of care debulking were obtained and processed within 24 h of surgery. Total tumor single cell suspensions were prepared using the GentleMACS kit (Miltenyi), then cryopreserved in 10% DMSO 90% human AB serum (Gemini) and stored in liquid nitrogen until use. Fresh ascites was processed within 24 h. Total ascites cells were isolated by centrifugation, red blood cells were removed by hypotonic lysis, and cells were cryopreserved in 10% DMSO 90% human AB serum and stored in liquid nitrogen until use. Tumor and ascites samples were obtained through the UHN Biospecimen Program and frozen plasma, and serum samples were obtained through the UHN GYN Blood Biobank. All patients gave informed written consent for specimen biobanking and research use. All protocols using human specimens were approved by the UHN Research Ethics Board.

### TIL expansion

Tumor-infiltrating or ascites-associated lymphocytes (TILs) were expanded from single cell suspensions isolated from primary surgical, paracentesis, or thoracentesis samples as previously described [38, 39]. Briefly, total tumor cells were cultured in IL-2 (3000–6000 IU/ml, Peprotech), in 24 well plates, and expanded for up to 4 weeks. Following initial expansion (primary TILs), TILs were assessed for CD3, CD4, and CD8 expression, cryopreserved in 10% DMSO 90% human AB serum, and stored in liquid nitrogen. Primary TILs were thawed and rapidly expanded with anti-CD3 stimulation and IL-2 on irradiated pooled donor PBMCs according to previously established protocols [38].

### Whole exome and transcriptome sequencing

Total tumor cells selected for genomic analyses were purified by magnetic sorting using CD45-beads (Miltenyi). For each patient, expanded TILs or the CD45 + fraction from whole tumor sorts were used as normal sequencing controls. DNA and RNA were co-isolated from purified cell pellets using Qiagen AllPrep Kits (QIAGEN). Whole exome sequencing was performed by Illumina HiSeq2500 using 125-cycle paired-end protocol and multiplexing to obtain 250 × coverage (tumor) or 50× coverage (normal), with Agilent SureSelect Human All Exon c5 + UTR library preparation. Transcriptome sequencing was performed using Illumina NextSeq500 with Illumina TruSeq Stranded Total RNA Sample Preparation Kit (RiboZero Gold), using 75-cycle paired end protocol for 80 M reads. Sample quality assessment was performed using BioAnalyser, TapeStation, and qPCR.

For initial samples in a pilot cohort, FASTQ files from WES were aligned to the human genome reference hg19 using the BWA-MEM [57] algorithm followed by Indel realignment and co-cleaning as outlined in the GATK best practices for exome sequencing [58] to generate BAM files for downstream analyses. Mutect v1.4 [59] was used to identify the somatic mutations from tumor and normal tissue BAM file pairs for each patient. Mutations with sufficient sequencing coverage and variant allele frequency greater and equal to 0.10 were selected for further analyses. RNA-seq FASTQs were aligned to the GRCh37 transcriptome reference and gencode v19 annotations using STAR v2.4.2a [60] with read length 100. BAM files were pre-processed according to the GATK RNA-seq short variant discovery best practices pipeline (GATK v3.0–0) with Picard MarkDuplicates, Indel realignment, and base recalibration prior to mutation calling [58]. Somatic mutations were detected using Mutect (v1.4) [59] and paired tumor and normal RNA-seq aligned bam files. We defined transcribed mutations as candidates found in both RNA-seq and WES derived mutation profiles for a single sample.

In the expanded cohort, WES and RNA-seq FASTQs were pre-processed following the same workflows as described in the pilot cohort. Genome reference hg38 and transcriptome reference GRCh38 were used for alignment. Due to contaminating reads from CD45-negative tumor cells in the normal reference samples, mutations from WES were identified using Mutect2 (GATK v3.8) [58] and RNA-seq were identified using GATK HaplotypeCaller (v3.5-0-g36282e4) [61] using tumor data as input. Using the bedtools v2.26.0 [62] intersect function, we selected mutations common to both WES and RNA-seq in each sequenced tumor sample after removing suspected germline SNPs (single nucleotide polymorphisms) with population allele frequency greater than 0.005 as reported by gnomAD v170228. All remaining variants were annotated with VEP v92 [63]. Gene-level transcript abundances were quantified using RSEM v1.2.29 [64], normalized across all samples and reported as TPM. HGSC gene expression data was validated by comparison to TCGA Ovarian Cancer cohost Illumina HiSEQ data, and all samples were found to have high pairwise Spearman correlations (0.885 overall).

Class I HLA type for each patient was determined using Seq2HLA [65] with RNA-seq FASTQ as input.

### Plasma auto-antibody profiling using ProtoArrays

Auto-antibody profiling was performed using ProtoArray 5.1 (Invitrogen) slides, according to the manufacturer’s instructions. Plasma was diluted 1:500 and detected using anti-human-IgG-Alexa-647. Slides were scanned on a Perkin Elmer ProScan Array scanner with 5 μm resolution, and images analyzed by ProtoArray Prospector software (Invitrogen) using Immune Response with Plasma parameters. Auto-antibodies against the known ovarian antigens MUC16, EPCAM, WT1, TP53, CTAG1A (NY-ESO-1) and FOLR1, could not be assessed in this assay due to their absence from the ProtoArray version 5.1 arrays. Significant auto-antibody “hits” are defined as those array targets having a signal greater than 1000 fluorescence units, and* Z*-Factor > 0.4.

*Z*-Factor is defined as:

$$Z = 1 - \frac{{3\sigma_{s} + 3\sigma_{c - } }}{{\left| {\mu_{s} - \mu_{c - } } \right|}}$$

where: *σs* = signal standard deviation for the target protein features; *σc-* = signal standard deviation for the negative control features; *μs* = mean signal for the target protein features; *μc-* = mean signal for the negative control features.

### Epitope prediction

Candidate target antigen amino acid sequences (canonical translation) from UniProt were examined for predicted HLA-A*02:01 binding affinity, proteasome processing, and TAP transport, using standalone NetCTLpan 1.1 software, querying 8, 9, and 10mers [31]. Epitopes with overall NetCTLpan scores > 0.8, NetMHCpan affinity < 500 nM, and acceptable hydrophobic residue content were considered for peptide synthesis and custom tetramer screening. Synthetic peptides were prepared by GenScript (Piscataway, NJ).

### Monocyte-derived dendritic cells

Monocytes were purified from healthy donor leukapheresis products by elutriation on an Elutra (Terumo BCT). Aliquots were viable frozen in 10% DMSO 90% human AB serum at 1 × 10^8^ cells/ml and stored in liquid nitrogen (vapor phase) until use. Cells were HLA typed by PCR and A*02:01 donor samples used to generate dendritic cells [66]. Briefly, monocytes were seeded in 6 well plates or T75 flasks in CellGro DC media (CellGenix) supplemented with 2% human AB serum and 50 μg/ml GM-CSF and 50 μg/ml IL-4, on day 3 half the media was replaced with fresh media and additional GM-CSF/IL-4. On day 5, cells were harvested by washing adherent DCs with PBS then incubating in Versene (Gibco) for 15 min at 37 °C, followed by gentle pipetting and centrifugation. Cell pellets were resuspended in freezing media, aliquoted, slow frozen, and stored in liquid nitrogen (vapor phase) until use.

### Peptide-specific TIL expansion and re-stimulation

Peptide-specific expansion of TILs was performed by culturing TILs in AIM-V media at 37 °C/5% CO_2_ with A2^+^ monocyte-derived dendritic cells (DC) that had been peptide pulsed overnight and matured with IL-1β (10 ng/ml), IL-6 (5 ng/ml), and TNFα (10 ng/ml) at a ratio of 10:1. Low dose IL-2 (200 IU/ml) was added with fresh media every 3 days for 10–14 days, splitting cultures as needed according to cell density. On day 14, cells were harvested, washed, treated with 2 μM brefeldin A, plated with mature monocyte-derived DC or T2 cells pulse with individual or pooled peptides, and incubated for 5 h at 37 °C 5/% CO_2_. Cells were then harvested, stained with anti-CD8 followed by fixation and permeabilization with Fix/Perm solution (BD Biosciences), then stained with anti-IFNγ in permeabilization solution (BD Bioscience). Cells were then washed, fixed in 2% paraformaldehyde (PFA)/PBS, and analysed by flow cytometry. To demonstrate endogenous processing and presentation of the candidate antigen epitopes by HGSC tumor cells, we first expanded the epitope-specific TIL populations with DC pulsed with individual peptides. On day 14, the expanded TILs were re-stimulated by co-culture with *unpulsed* epithelial tumor cells grown from the CD45-negative fraction of matched patient tumor samples, in the presence of brefeldin A, and IFNγ production assessed as described above.

### Custom peptide-MHC (pMHC) tetramers and TIL staining

Monomeric HLA-A*02:01 loaded with UV-sensitive peptide (KILGFVFJV) was purchased from BioLegend (UVX-A0201). Custom exchanged monomers were produced as described [67]. Briefly, 100 × excess molar ratio of custom peptide was added to UV-sensitive monomers followed by exposure to long wave UV light (352 nm) for 60 min on ice. The reaction product was centrifuged at 3500×*g* for 5 min to remove unassembled empty HLA precipitates, and tetramers assembled with Steptavidin-PE (Invitrogen) at a 4:1 molar ratio. Tetramers were stored at 4 °C until use. For detection of peptide-specific T cells, expanded cultures were treated with dasatinib (5 nM) for 30 min at 37 °C, and the cells harvested, resuspend in PBS with 2% FBS, and stained with custom tetramers (5 μl per 2 × 10^6^ cells). Following incubation for 20 min at room temperature, anti-CD8 and viability dye were added, and the incubation continued for 15 min at 4 °C. Cells were then washed, fixed in 2% PFA/PBS, and analysed by flow cytometry.

### Mass spectrometry analyses

Primary ovarian tumor samples, either viable cryopreserved tumor surgical resection fragments (OV606), or magnetic bead sorted, CD45-negative ascites cells (OV633, OV642), were used as starting material. Approximately 1 g solid tumor or 1 × 10^8^ sorted tumor cells were processed for HLA precipitation and peptide elution as described [44]. Liquid chromatography-tandem mass spectrometry (LC–MS/MS) analyses of the corresponding peptide extracts were acquired on a Q-Exactive HF mass spectrometer (Thermo Fisher Scientific) with parameter settings as defined previously [44]. All MS data were search against a patient-specific cancer database using using PEAKS X (Bioinformatics Solutions Inc), with tolerance and threshold setting as previously described [44].

## Supplementary Information

Below is the link to the electronic supplementary material.

Supplementary file1 (PDF 33581 KB)

## Data Availability

All raw ProtoArray data, processed RNA-seq data, and gene sets from this study are available upon reasonable request. Mass spectrometry raw data were deposited to the ProteomeXchange Consortium via the PRIDE partner repository with the following data set identifiers: PXD014062 and 10.6019/PXD014062.

## References

[1] Andersen RS, Thrue CA, Junker N (2012). Dissection of T-cell antigen specificity in human melanoma. Cancer Res.

[2] Laumont CM, Vincent K, Hesnard L et al (2018) Noncoding regions are the main source of targetable tumor-specific antigens. Sci Transl Med 10. 10.1126/scitranslmed.aau5516 10.1126/scitranslmed.aau5516 30518613

[3] Ouspenskaia T, Law T, Clauser KR (2022). Unannotated proteins expand the MHC-I-restricted immunopeptidome in cancer. Nat Biotechnol.

[4] Schumacher T, Bunse L, Pusch S (2014). A vaccine targeting mutant IDH1 induces antitumour immunity. Nature.

[5] Tran E, Robbins PF, Lu YC (2016). T-cell transfer therapy targeting mutant KRAS in cancer. N Engl J Med.

[6] Deniger DC, Pasetto A, Robbins PF (2018). T-cell responses to TP53 "Hotspot" mutations and unique neoantigens expressed by human ovarian cancers. Clin Cancer Res.

[7] Schumacher TN, Scheper W, Kvistborg P (2019). Cancer neoantigens. Annu Rev Immunol.

[8] Laureano RS, Sprooten J, Vanmeerbeerk I (2022). Trial watch: Dendritic cell (DC)-based immunotherapy for cancer. Oncoimmunology.

[9] Robbins PF, Lu Y-C, El-Gamil M (2013). Mining exomic sequencing data to identify mutated antigens recognized by adoptively transferred tumor-reactive T cells. Nat Med.

[10] van Rooij N, van Buuren MM, Philips D (2013). Tumor exome analysis reveals neoantigen-specific T-cell reactivity in an ipilimumab-responsive melanoma. J Clin Oncol.

[11] Lu YC, Yao X, Li YF (2013). Mutated PPP1R3B is recognized by T cells used to treat a melanoma patient who experienced a durable complete tumor regression. J Immunol.

[12] Tran E, Turcotte S, Gros A (2014). Cancer immunotherapy based on mutation-specific CD4+ T cells in a patient with epithelial cancer. Science.

[13] Zacharakis N, Chinnasamy H, Black M (2018). Immune recognition of somatic mutations leading to complete durable regression in metastatic breast cancer. Nat Med.

[14] Carreno BM, Magrini V, Becker-Hapak M (2015). A dendritic cell vaccine increases the breadth and diversity of melanoma neoantigen-specific T cells. Science.

[15] Ott PA, Hu Z, Keskin DB (2017). An immunogenic personal neoantigen vaccine for patients with melanoma. Nature.

[16] Sahin U, Derhovanessian E, Miller M (2017). Personalized RNA mutanome vaccines mobilize poly-specific therapeutic immunity against cancer. Nature.

[17] Keskin DB, Anandappa AJ, Sun J (2019). Neoantigen vaccine generates intratumoral T cell responses in phase Ib glioblastoma trial. Nature.

[18] Torre LA, Trabert B, DeSantis CE, Miller KD, Samimi G, Runowicz CD, Gaudet MM, Jemal A, Siegel RL (2018). Ovarian cancer statistics, 2018. CA Cancer J Clin.

[19] Varga A, Piha-Paul S, Ott PA, Mehnert JM, Berton-Rigaud D, Morosky A, Yang P, Ruman J, Matei D (2019). Pembrolizumab in patients with programmed death ligand 1-positive advanced ovarian cancer: Analysis of KEYNOTE-028. Gynecol Oncol.

[20] Clouthier DL, Lien SC, Yang SYC (2019). An interim report on the investigator-initiated phase 2 study of pembrolizumab immunological response evaluation (INSPIRE). J Immunother Cancer.

[21] TCGA (2011). Integrated genomic analyses of ovarian carcinoma. Nature.

[22] Martin SD, Wick DA, Nielsen JS, Little N, Holt RA, Nelson BH (2017) A library-based screening method identifies neoantigen-reactive T cells in peripheral blood prior to relapse of ovarian cancer. Oncoimmunology 7:e1371895. 10.1080/2162402X.2017.1371895 10.1080/2162402X.2017.1371895 PMC5739566 29296522

[23] Bobisse S, Genolet R, Roberti A (2018). Sensitive and frequent identification of high avidity neo-epitope specific CD8 (+) T cells in immunotherapy-naive ovarian cancer. Nat Commun.

[24] Cafri G, Yossef R, Pasetto A (2019). Memory T cells targeting oncogenic mutations detected in peripheral blood of epithelial cancer patients. Nat Commun.

[25] Liu S, Matsuzaki J, Wei L (2019). Efficient identification of neoantigen-specific T-cell responses in advanced human ovarian cancer. J Immunother Cancer.

[26] Sahin U, Tureci O, Schmitt H (1995). Human neoplasms elicit multiple specific immune responses in the autologous host. Proc Natl Acad Sci U S A.

[27] Bevan MJ (2004). Helping the CD8(+) T-cell response. Nat Rev Immunol.

[28] Jäger E, Chen YT, Drijfhout JW (1998). Simultaneous humoral and cellular immune response against cancer-testis antigen NY-ESO-1: definition of human histocompatibility leukocyte antigen (HLA)-A2-binding peptide epitopes. J Exp Med.

[29] Milne K, Barnes RO, Girardin A et al (2008) Tumor-infiltrating T cells correlate with NY-ESO-1-specific autoantibodies in ovarian cancer. PLoS One 3:e3409. 10.1371/journal.pone.0003409 10.1371/journal.pone.0003409 PMC2561074 18923710

[30] Hulett TW, Jensen SM, Wilmarth PA, Reddy AP, Ballesteros-Merino C, Afentoulis ME, Dubay C, David LL, Fox BA (2018). Coordinated responses to individual tumor antigens by IgG antibody and CD8+ T cells following cancer vaccination. J Immunother Cancer.

[31] Stranzl T, Larsen MV, Lundegaard C, Nielsen M (2010). NetCTLpan: pan-specific MHC class I pathway epitope predictions. Immunogenetics.

[32] Wells DK, van Buuren MM, Dang KK et al (2020) Key parameters of tumor epitope immunogenicity revealed through a consortium approach improve neoantigen prediction. Cell 183:818–34 e13. 10.1016/j.cell.2020.09.015 10.1016/j.cell.2020.09.015 PMC7652061 33038342

[33] Nagele EP, Han M, Acharya NK, DeMarshall C, Kosciuk MC, Nagele RG (2013) Natural IgG autoantibodies are abundant and ubiquitous in human sera, and their number is influenced by age, gender, and disease. PLoS One 8:e60726. 10.1371/journal.pone.0060726 10.1371/journal.pone.0060726 PMC3617628 23589757

[34] Gnjatic S, Ritter E, Buchler MW (2010). Seromic profiling of ovarian and pancreatic cancer. Proc Natl Acad Sci U S A.

[35] Pinto S, Michel C, Schmidt-Glenewinkel H, Harder N, Rohr K, Wild S, Brors B, Kyewski B (2013). Overlapping gene coexpression patterns in human medullary thymic epithelial cells generate self-antigen diversity. Proc Natl Acad Sci U S A.

[36] Olsen LR, Tongchusak S, Lin H, Reinherz EL, Brusic V, Zhang GL (2017). TANTIGEN: a comprehensive database of tumor T cell antigens. Cancer Immunol Immunother.

[37] Hoadley KA, Yau C, Wolf DM (2014). Multiplatform analysis of 12 cancer types reveals molecular classification within and across tissues of origin. Cell.

[38] Nguyen LT, Yen PH, Nie J et al (2010) Expansion and characterization of human melanoma tumor-infiltrating lymphocytes (TILs). PLoS One 5:e13940. 10.1371/journal.pone.0013940 10.1371/journal.pone.0013940 PMC2978109 21085676

[39] Crome SQ, Nguyen LT, Lopez-Verges S (2017). A distinct innate lymphoid cell population regulates tumor-associated T cells. Nat Med.

[40] Murata K, Nakatsugawa M, Rahman MA et al (2020) Landscape mapping of shared antigenic epitopes and their cognate TCRs of tumor-infiltrating T lymphocytes in melanoma. Elife, 9. 10.7554/eLife.53244 10.7554/eLife.53244 PMC7234812 32314731

[41] Brown SD, Raeburn LA, Holt RA (2015). Profiling tissue-resident T cell repertoires by RNA sequencing. Genome Med.

[42] Simoni Y, Becht E, Fehlings M (2018). Bystander CD8(+) T cells are abundant and phenotypically distinct in human tumour infiltrates. Nature.

[43] Scheper W, Kelderman S, Fanchi LF (2019). Low and variable tumor reactivity of the intratumoral TCR repertoire in human cancers. Nat Med.

[44] Zhao Q, Laverdure JP, Lanoix J (2020). Proteogenomics uncovers a vast Repertoire of shared tumor-specific antigens in ovarian cancer. Cancer Immunol Res.

[45] Schuster H, Peper JK, Bosmuller HC (2017). The immunopeptidomic landscape of ovarian carcinomas. Proc Natl Acad Sci U S A.

[46] Tang Z, Li C, Kang B, Gao G, Li C, Zhang Z (2017). GEPIA: a web server for cancer and normal gene expression profiling and interactive analyses. Nucleic Acids Res.

[47] Rodriguez-Garcia A, Minutolo NG, Robinson JM, Powell DJ (2017). T-cell target antigens across major gynecologic cancers. Gynecol Oncol.

[48] Kreiter S, Vormehr M, van de Roemer N (2015). Mutant MHC class II epitopes drive therapeutic immune responses to cancer. Nature.

[49] Linnemann C, van Buuren MM, Bies L (2015). High-throughput epitope discovery reveals frequent recognition of neo-antigens by CD4+ T cells in human melanoma. Nat Med.

[50] Winter SF, Minna JD, Johnson BE, Takahashi T, Gazdar AF, Carbone DP (1992). Development of antibodies against p53 in lung cancer patients appears to be dependent on the type of p53 mutation. Cancer Res.

[51] Gjerstorff MF, Andersen MH, Ditzel HJ (2015) Oncogenic cancer/testis antigens: prime candidates for immunotherapy. Oncotarget 6:15772–15787. 10.18632/oncotarget.4694 10.18632/oncotarget.4694 PMC4599236 26158218

[52] Bezu L, Kepp O, Cerrato G, Pol J, Fucikova J, Spisek R, Zitvogel L, Kroemer G, Galluzzi L (2018) Trial watch: Peptide-based vaccines in anticancer therapy. Oncoimmunology 7:e1511506. 10.1080/2162402X.2018.1511506 10.1080/2162402X.2018.1511506 PMC6279318 30524907

[53] Yadav M, Jhunjhunwala S, Phung QT (2014). Predicting immunogenic tumour mutations by combining mass spectrometry and exome sequencing. Nature.

[54] Zitvogel L, Perreault C, Finn OJ, Kroemer G (2021). Beneficial autoimmunity improves cancer prognosis. Nat Rev Clin Oncol.

[55] Meunier MC, Delisle JS, Bergeron J, Rineau V, Baron C, Perreault C (2005). T cells targeted against a single minor histocompatibility antigen can cure solid tumors. Nat Med.

[56] Nathan P, Hassel JC, Rutkowski P (2021). Overall survival benefit with Tebentafusp in metastatic uveal melanoma. N Engl J Med.

[57] Li H (2013) Aligning sequence reads, clone sequences and assembly contigs with BWA-MEM. arXiv. 1303.3997. http://arxiv.org/abs/1303.3997

[58] Van der Auwera GA, Carneiro MO, Hartl C et al (2013) From FastQ data to high confidence variant calls: the Genome Analysis Toolkit best practices pipeline. Curr Protoc Bioinformatics 43:1101–1133. 10.1002/0471250953.bi1110s43 10.1002/0471250953.bi1110s43 PMC4243306 25431634

[59] Cibulskis K, Lawrence MS, Carter SL (2013). Sensitive detection of somatic point mutations in impure and heterogeneous cancer samples. Nat Biotechnol.

[60] Dobin A, Davis CA, Schlesinger F, Drenkow J, Zaleski C, Jha S, Batut P, Chaisson M, Gingeras TR (2013). STAR: ultrafast universal RNA-seq aligner. Bioinformatics.

[61] Poplin R, Ruano-Rubio V, DePristo MA et al (2017) Scaling accurate genetic variant discovery to tens of thousands of samples. bioRxiv. 201178. 10.1101/201178

[62] Quinlan AR, Hall IM (2010). BEDTools: a flexible suite of utilities for comparing genomic features. Bioinformatics.

[63] McLaren W, Gil L, Hunt SE, Riat HS, Ritchie GR, Thormann A, Flicek P, Cunningham F (2016). The ensemble variant effect predictor. Genome Biol.

[64] Li B, Dewey CN (2011). RSEM: accurate transcript quantification from RNA-seq data with or without a reference genome. BMC Bioinformatics.

[65] Boegel S, Lower M, Schafer M (2012). HLA typing from RNA-seq sequence reads. Genome Med.

[66] Scheid E, Major P, Bergeron A (2016). Tn-MUC1 DC v of Rhesus Macaques and a phase I/II trial in patients with nonmetastatic Castrate-resistant prostate cancer. Cancer Immunol Res.

[67] Rodenko B, Toebes M, Hadrup SR, van Esch WJE, Molenaar AM, Schumacher TNM, Ovaa H (2006). Generation of peptide-MHC class I complexes through UV-mediated ligand exchange. Nat Protoc.

