# Supplementary material for: Identification of antigenic epitopes recognized by tumor infiltrating lymphocytes in high grade serous ovarian cancer by multi-omics profiling of the auto-antigen repertoire
Source: Cancer Immunol Immunother. 2023 Mar 21;72(7):2375–92. doi: 10.1007/s00262-023-03413-7 (PMC10264507; doi:10.1007/s00262-023-03413-7)

Supplementary Tables and Figures

Supplementary Table S1. Patient Characteristics and Summary of Auto-antibodies. Auto-antibody “Hits” were defined as genes on the ProtoArrays with Z-Score > 0.4 and signal intensity > 1000. *HGS*, high grade serous carcinoma; *HNSCC*, head and neck squamous cell carcinoma; *MEL*, metastatic melanoma; *Sarcoma FT*, sarcoma fibrous tumor; *CEAD*, colonic epithelial adenocarcinoma; *Merkel*, Merkel cell carcinoma.

Patient ID	Age	Sex	Pathology	Auto-antibody “Hits”
OV158	51	F	HGS, Primary	1274
OV237	63	F	HGS, Recurrence	850
OV248	59	F	HGS, Recurrence	734
OV355	75	F	HGS, Primary	1301
OV364	77	F	HGS, Recurrence	276
OV436	80	F	HGS, Primary	640
OV486	72	F	HGS, Primary	299
OV499	74	F	HGS, Primary	200
OV586	58	F	HGS, Recurrence	354
Mean	67.7			659

Patient ID	Age	Sex	Pathology	Auto-antibody “Hits”
S-001	62	M	HNSCC	21
S-002	70	M	HNSCC	643
S-003	73	M	MEL	213
S-004	67	F	MEL	165
S-005	57	M	MEL	976
S-006	74	M	MEL	1549
S-007	42	M	Sarcoma FT	47
S-008	44	F	CEAD	158
S-009	81	M	Merkel	100
Mean	63.3			430

Supplementary Table S2. HLA-A*02:01 epitope peptide pools used in this study. **(a)** Seven peptides from viral pathogens (HIV, CMV, EBV, and Flu, and Vaccinia), used as internal negative and positive controls. **(b)** Twenty-nine known tumor-associated antigen (TAA) epitope peptides selected from the TANTIGEN database. **(c)** Twenty-four predicted epitopes from HGSC patient shared auto-antibody target proteins. **(d)** The sequences of each SNV neo-epitope chosen from each HGSC patient, with their associated NetCTLpan and NetMHCpan scores for HLA-A*02:01 are shown.

Supplementary Table S2.

a. Control CEF epitopes

Gene	Position	Sequence	NetCTLpan (Score)	NetMHCpan (Affinity)
HIV pol	476-484	ILKEPVHGV	0.87902	42
Flu M1	58-66	GILGFVFTL	0.93465	29
Flu PA	46-54	FMYSDFHFI	1.16574	1.9
EBV LMP2a	426-434	CLGGLLTMV	0.75644	90
EBV BMLF1	280-288	GLCTLVAML	0.78363	101
HCMV pp65	495-503	NLVPMVATV	0.85518	58
Vacc J8R	391-399	AMLNGLIYV	1.08678	2.4

b. 29 known HLA –A*02:01 TAA epitopes

Gene	Position	Sequence	NetCTLpan (Score)	NetMHCpan (Affinity)
WT1	126	RMFPNAPYL	1.06459	8.2
MUC16	11190	KMISAIPTL	1.12516	4.0
CRABP1	60	RTTEINFKV	0.70803	289.9
MAGEA3/A12	220	KIWEELSVL	0.84728	81.7
MAGEA10	254	GLYDGMEHL	1.09388	6.0
MAGEB1/B2	270/273	FLWGPRAYA	0.95507	13.1
BIRC5	5	TLPPAWQPFL	0.86114	56.6
BIRC5	96 T97M	LMLGEFLKL	1.2425	26.1
ERBB2	369	KIFGSLAFL	0.99297	16.7
ERBB2	435	ILHNGAYSL	0.87388	57.8
MSLN	20	SLLFLLFSL	1.01659	11.5
MSLN	539	VLPLTVAEV	0.89221	40.0
MSLN	549	KLLGPHVEGL	0.94563	25.4
FOLR1	191	EIWTHSYKV	0.8068	473.8
NY-ESO-1	157 C165V	SLLMWITQV	1.4229	5.4
PRAME	142	SLYSFPEPEA	0.78384	51.9
PRAME	300	ALYVDSLFFL	1.01753	12.2
PRAME	425	SLLQHLIGL	0.90499	17.6
BIRC7	34	SLGSPVLGL	0.66842	503.4
BIRC7	90	RLASFYDWPL	1.07203	6.1
CDK1	134	LLIDDKGTIKL	0.88719	53.8
ETV5	45	ELFQDLSQL	0.55041	1009.6
MDM2	48	YTMKEVLFYL	1.07748	4.4
MDM2	53	VLFYLGQYI	0.65813	100.4
MDM2	80	DLLGDLFGV	0.81293	30.9
MDM2	81	LLGDLFGV	1.04486	6.4
STAT1	350	KLQELNYNL	1.03369	7.1
BCAP31	167	KLDVGNAEV	0.95214	19.2
EPCAM	263	GLKAGVIAV	0.71942	44.6

Supplementary Table S2. Continued.

C. 24 predicted HLA-A*02:01 epitopes from shared Auto-Antibody targets

Gene	Position	Sequence	NetCTLpan (Score)	NetMHCpan (Affinity)
MOB1A	117	YLMTWVQDQL	1.1351	2.9
MOB1A	130	TLFPSKIGV	0.89665	28.3
MOB1A	70	MLYGTITEFC	0.70941	554.9
EIF4H	105	LLGDRSLRV	0.80142	97.2
SOCS3	177	VLSRPLSSNV	0.81745	54.2
STAM	374	LMNEDPMYSM	0.97561	14.5
ABCF3	459	KMLEKLPEL	1.1503	2.9
KIAA1217	1385	FMITETTVQV	1.12425	3.2
LARP7	368	KMGEEVIPL	1.01405	10.4
FTH1	138	YLNEQVKAI	0.90779	27.1
TUBB	206	ALYDICFRTL	0.93179	29.6
PRKAR1A	294	IILEGSAAV	1.02806	8.1
CCDC6	369	GLSYASHTV	0.92133	25.1
BRD9	362	KLLPGFTTL	1.06705	7.2
HSBP1	55	LMTQAGVEEL	0.77462	151.5
SDCCAG8	588	LLTSQNTFL	0.84322	70.3
CAB39L	236	ILDRHNFAI	0.88524	33.3
EBAG9	14	CLATVFSFL	0.90815	26.8
FLOT2	398	LLAELPASV	1.0719	3.9
RAC1	35	TVFDNYSANV	0.90166	32.6
RAC1	144	AMAKEIGAV	0.87611	18.6
DDIT4	200	FLPGFSQSL	1.0018	12.9
AES	140	LPLTPLPV	0.93661	28.6
PSMB4	189	YLAQPLLREV	0.99402	5.0

Supplementary Table S2. Continued

d. 48 predicted HLA-A*02:01 neo-epitopes from 9 HGSC patients

Gene	Mutation	Epitope	NetCTLpan (Score)	NetMHCpan (Affinity)	Patient ID
C9orf89	R61L	VLLCDLLSHL	0.97714	19	OV158
TP53	V216M	MVVPYEPPEV	0.81025	58.5	OV158
CBFA2T2	C131W	VTIEEFHWKL	0.70797	341	OV237
CBL	E111K	TLGKNEYFRV	0.65005	75.8	OV237
POP1	A280T	GLTFATVHCL	0.79144	126	OV237
SLC2A1	Q25P	SLPFGYNTGV	0.89168	37.1	OV237
SMARCA4	T910M	RLLLMGTPL	0.74278	211.8	OV237
TAF1	V1545L	ILNPMDLETI	0.65355	64.4	OV237
DHX57	I1213L	LLSAMLCAAL	0.72055	21.1	OV248
SCN8A	K127T	AITILIHSV	0.66935	172.5	OV248
CARD11	K1019M	FLRRQMTETI	0.63559	375.9	OV248
ATM	F858L	LLNDYPDSSV	0.92012	16.1	OV355
RAD1	E124G	FLGEGGVVTV	1.04633	5.3	OV355
DNAJB12	M266I	ILVSALSQLI	0.75706	71.8	OV364
ENAH	A103V	SVMMHALEV	0.81063	87.2	OV364
PPP1R12C	V304L	ELLSLLEEL	0.73191	228.5	OV364
TCP11X2	R223W	LLMDRIWLQ	0.65839	108.3	OV436
IDH2	T435M	KLNEHFLNTM	0.83381	28.9	OV436
STXBP5	V337M	AMLEMDYSI	0.90955	5.1	OV436
MT-ND4L	V59M	IMPIAMLVFA	0.51138	246.5	OV436
ATR	M211T	TLLTVLTRI	0.69198	47.1	OV436
TP53	R249G	GMNRGPILTI	0.69184	286.8	OV436
SMPDL3A	K369M	WMLEYILTQT	0.79898	9.5	OV486
IDE	Q399L	ILHMFQYIL	0.71837	299.5	OV486
PML	F645L	ALFSIYSKAV	0.90444	19	OV486
CFAP43	E1642K	KQISILQTEV	0.91823	23.6	OV486
TNK2	S607F	SLLDETPPQF	0.71514	461.7	OV486
HIST1H2BH	F71L	IMNSFVNDIL	0.71253	127.4	OV486
CCDC14	R906T	RLQKSLTTGL	0.71397	302.7	OV486
PRKDC	V417F	LQSFASVLL	0.67719	446.9	OV486
GGT7	A167V	SVDAVVAAA	0.62297	236	OV486
RNF213	P729L	ALEGLSFSL	0.84593	77.4	OV499
TTC30B	P317A	KLQFLLQQNA	0.50704	280.6	OV499
EPS15	I822M	EMFCDPFTSA	0.63376	316.1	OV499
NUP214	M572V	SVSAPNIAV	0.67104	401.1	OV499
ATR	R2425Q	KLKVFQEFL	0.66312	461.7	OV499
PLAA	E363G	KVGAYQWSV	0.92228	19.4	OV499
KDELR2	L161F	FLGLYRAFYL	0.79153	14.8	OV499
KIAA1468	L1201F	FLNKMGQFTT	0.53526	148.2	OV499
TMTC2	F233L	ILWGSSLLGA	0.6573	42.3	OV586
FAT4	Q453L	VLARSSVASL	0.79988	54.2	OV586
BARD1	V507M	HMDIVKLLL	0.71343	265.9	OV586
GOSR1	M70V	RMFETVAIEI	0.92088	13.1	OV586
HECTD4	E3862Q	QLRTCGLPYI	0.61858	471.8	OV586
GMEB1	E228D	GLDWNSALTA	0.61944	289.9	OV586
NSD1	V614L	SLLCGSKVKL	0.7357	221.2	OV586
FGFR1OP	T204I	NQSDISVSL	0.69751	396.8	OV586
STX2	R124G	VLSGKFVEA	0.79205	84.4	OV586

Supplementary Table S3. SNV in 9 HGSC patients. Expressed somatic single nucleotide variants from combined WES and RNA-seq data were determined as described in Materials and Methods. For each identified mutation, the gene symbol and non-synonymous amino acid substitution is shown.

Supplementary Table 3.

OV158	OV237	OV248	OV355	OV364	OV436	OV486	OV499	OV586
ASCC1, R109T	ADRBK2, K628N	ARID2, A1434S	ATM, F858L	CNOT10, D102H	ACSL3, G239E	ARFGEF1, Q630H	ATR, R2425Q	APOBEC3B, T146K
BID, L103P	AKAP9, V1571M	BUB1B, R349Q	BCL9, P671S	DNAJB12, M266I	ASXL1, E1102D	ARHGEF12, Y973F	BCORL1, G209S	ARHGEF12, Y1039H
C9orf89, R61L	CBFA2T2, C131W	CARD11, K1019M	HSPBAP1, S372N	ENAH, A103V	ATPAF1, R213W	BCL6, A493T	CLIP1, A1224S	ARNT, R222H
CAMSAP1, A927T	CBL, E111K	CCNH, Q142H	JUN, S267C	GGNBP2, L122M	ATR, M211T	C16orf58, T215S	DLGAP1, D579H	BARD1, V507M
CCNT2, S537C	CDC34, E235D	CHD8, E1771Q	MECOM, P120S	HIST1H2AE, E57K	C2CD2, V296A	CCDC14, R906T	DUS4L, K39E	C5orf15, P56Q
IFI44, E66G	CEP192, I1858V	DHX57, I1213L	MYC, N26S	PPP1R12C, V304L	CBLC, P435S	CCND3, S259A	EPS15, I822M	C17orf51, A106G
KDM1A, G837A	CHD2, T1502S	EFHD2, R91M	MYO5A, R1246C	RNPEPL1, S469L	CCNT1, R122G	CD109, R663S	FANCE, A502T	CD24, A106V
LARP6, G254E	EIF2B3, C300S	EZH2, D185H	RAD1, E124G	TNFAIP8, E60K	CDKN1B, V109G	CFAP43, E1642K	HSD17B2, A224S	CERS5, W167L
MTO1, R91K	ELP3, H190Y	FAM200B, H545N	SESTD1, E635K	TTC14, P587S	CIPC, M63V	CSMD2, G2010E	KDELR2, L161F	CREBZF, K314N
PTBP3, K188R	FASN, N2033S	FGFR2, N550K	SPEN, L1091P	ZNF93, G109V	DDX5, S480A	DIXDC1, E294V	KIAA1468, L1201F	EPAS1, T766P
TP53, V216M	FBXO34, C174G	IPO5, K1077R	TET2, I1762V		DICER1, A872T	DNPH1, P18T	KRAS, G13D	EPHB4, T210I
	FLNA, P2354A	LIFR, D578N	TSC1, M322T		EML6, S797N	ELL, A74V	LIFR, D1049V	FAM107A, P126S
	FLNB, G2042S	MLLT10, K318R	ZBTB7B, Q12E		EP300, I997V	ERC1, S50G	MAML2, P896T	FAT4, Q453L
	GON4L, E1496A	MT-CO1, S101N			ETV1, S100G	ERCC4, R415Q	MAP3K1, T542P	FGFR1OP, T204I
	KDM4B, P744L	MTHFS, L104F			FANCG, R513Q	EXT2, I159V	MT-CO1, F293S	FMNL3, E427Q
	LTA4H, Q499E	NBPF19, D3796E			FGFR3, P451S	FBXO41, R858Q	MYC, F22L	GMEB1, E228D
	MAST2, S1270I	NOTCH2, G1016S			FOSL2, E123G	FGD5, G728V	MYO1D, F747I	GORASP2, S292F
	PATZ1, R454Q	NT5C2, T3A			FOXO1, V389I	FTSJ1, K62R	NCOA2, M1282I	GOSR1, M70V
	PCNXL2, Y1196F	P2RY12, T126I			HERPUD1, R50H	GALNS, L91V	NFX1, H462D	HECTD4, E3862Q
	POP1, A280T	PIK3R1, M326I			IDH2, T435M	GATA2, A164T	NUP214, M572V	ITGB6, C41R
	PPDPF, G37V	PPA1, R177Q			LRIG1, N219K	GGT7, A167V	PALB2, Q559R	KDM6A, T726K
	SLC2A1, Q25P	SCN8A, K127T			LRPPRC, K1020T	GRIPAP1, L179P	PIGU, L248V	KMT2C, S3660L
	SMARCA4, T910M	SDC4, F12L			MET, T733I	HIST1H2BH, F71L	PLAA, E363G	LZTR1, R440G
	TAF1, V1545L	SPEN, A970V			MT-CO2, G169S	HIST1H3E, T7M	POLI, Y127H	MLLT10, T835I
	TP53, R273H	SRGAP3, I371L			MT-ND4L, V59M	HIVEP1, H945R	POLQ, Q2513R	NBPF14, S1521G
	TRIT1, K417R	TCF4, S483F			NCAM2, A98T	HSPA9, D277N	PRDM1, D203E	NBPF19, R332H
	VPS13B, I667T	TGFBR2, R19L			NME5, I10M	IDE, Q399L	PRIM1, N62K	NECTIN4, E389Q
	ZMYM4, K583E	TNFRSF14, K17R			NPIPB6, D380Y	KAT6A, E1412K	RAB3D, S32N	NFATC2, H446R
	ZNF200, R372K	TRIP11, G1827S			NTN4, P139A	KIAA1456, Q11L	RNF213, P729L	NFXL1, H454R
	ZNF407, N765S	ZNF208, G897A			NUP98, Q1142E	LIFR, H116Y	RSF1, T812A	NPAT, L768P
		ZNF606, T449A			NUP205, V1053M	MLF1, P257T	SDHA, A454T	NSD1, V614L
		ZNF773, F184L			PLCG1, S279G	NBPF11, Y680C	TMEM236, N259K	NUB1, E512G
					PRELID3B, K48R	NBPF12, A129T	TP53, S241Y	NUP155, V658A
					PTCH1, P725S	NBPF14, D442N	TRIP10, P396S	PBX1, G21S

OV158	OV237	OV248	OV355	OV364	OV436	OV486	OV499	OV586
					RNF41, E14K	NBPF26, T235S	TTC30B, P317A	PEX26, H278Y
					RNF43, R117H	NCOR2, A1699T	UBR5, K2063N	PLS1, K471E
					RPL4, R45C	NUMA1, K374R	ULK2, T555I	PML, A858V
					RPS3A, Q163E	OSBPL2, K150E	XRCC4, L305P	POLE, A1778T
					RRP9, R466S	PML, F645L		POLK, N518S
					SETBP1, V1101I	PMS2, G857A		POLQ, A2547V
					SLC39A4, V372L	POM121, L985P		RBP4, L140R
					STXBP5, V337M	PPARG, P12A		RNF213, Q1133K
					SUPT20H, P420L	PRCC, P136S		SDC4, F12L
					SUZ12, A66T	PRKDC, V417F		SPANXB1, L74V
					TCP11X2, R223W	PRPS2, M73I		STX2, R124G
					TP53, R249G	PRSS48, C53R		TCF7L2, P483T
					TRRAP, V2301I	PTPRB, S345G		TERF2IP, L7V
					XAB2, G247C	RABEP1, M628I		TET2, V218M
						RECQL4, R1005Q		TMTC2, F233L
						RNF43, I47V		TRIP11, E1696K
						SALL4, L507R		URB1, M2013I
						SH2B1, S200C		ZNF318, G1906V
						SLC34A2, D634G		ZNF331, C85R
						SMPDL3A, K369M		ZSCAN2, L124Q
						SRGAP2C, H235R		
						SRRM2, D267G		
						TARS, V659L		
						THBS1, T221A		
						THSD7A, S1583F		
						TMEM205, N6D		
						TNK2, S607F		
						TPR, S960N		
						VCP, V176A		
						XRN2, H227P		
						ZNF34, G153E		
						ZNF670, Y309C		
						ZNF718, T23I		

Supplementary Figure S1. Comparison of HGSC and healthy donor auto-antibody repertoires. (**a**) Gene family analysis (https://www.gsea-msigdb.org/gsea/msigdb/gene_families.jsp) was performed on the 379 shared auto-antibody targets in healthy donors (reported in Ref.[33]). (**b**) Gene family analysis of the 524 commonly shared and differentially enriched auto-antibody targets in 9 HGSC patients from this study. **(c)** List of the ten cytokine and growth factor gene targets of auto-antibodies unique to HGSC patients, identified from panel **b**. (**d)** Venn diagram showing overlap of the 524 shared auto-antibody target genes from HGSC patients (this study) versus 751 auto-antibody targets in breast cancer (BC) patients and 620 common targets of healthy donors (HD) (reported in Ref. [33]).

Supplementary Figure S1.

a Gene families of auto-antibody targets common in Healthy Donors

	cytokines and growth factors	transcription factors	homeodomain proteins	cell differentiation markers	protein kinases	translocated cancer genes	oncogenes	tumor suppressors
tumor suppressors	0	0	0	0	0	0	0	2
oncogenes	0	3	0	1	3	9	10	
translocated cancer genes	0	3	0	0	3	9		
protein kinases	0	1	0	0	28			
cell differentiation markers	0	0	0	7				
homeodomain proteins	0	5	5					
transcription factors	0	31						
cytokines and growth factors	0							

b Gene families of auto-antibody targets in HGSC patients

	cytokines and growth factors	transcription factors	homeodomain proteins	cell differentiation markers	protein kinases	translocated cancer genes	oncogenes	tumor suppressors
tumor suppressors	0	1	1	1	0	0	0	3
oncogenes	0	3	0	0	1	11	11	
translocated cancer genes	0	3	0	0	1	12		
protein kinases	0	0	0	0	33			
cell differentiation markers	1	0	0	11				
homeodomain proteins	0	5	5					
transcription factors	0	41						
cytokines and growth factors	10							

c

Cytokine and Growth Factor targets of auto-antibodies
CD40LG
CGB1
CMTM1
CNTF
CXCL11
CXCL2
FGF1
FGF12
IFNG
MSTN

d

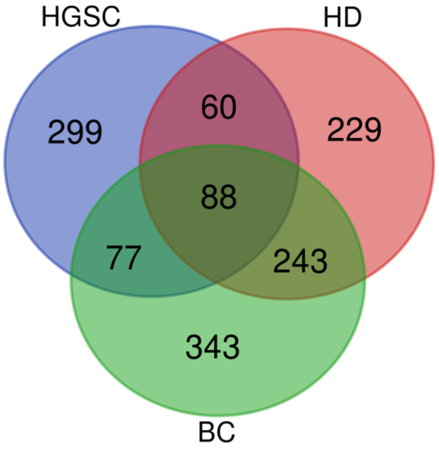

Supplementary Figure S2. Elevated occurrence of auto-antibodies targeting highly expressed and ovarian cancer-enriched genes in HGSC patients compare to non-HGSC and healthy donors. **(a)** ProtoArray Z-Factors for the top 160 TCGA OV (ovarian cancer) PANCAN enriched genes were extracted for 9 patients (different symbols) in each of 3 groups, HGSC patients, non-HGSC patients, and Healthy donors. The vertical line represents the cut-off value for a significant auto-antibody "hit" (0.4). **(b)** HGSC shared auto-antibody target genes (red symbols) were examined for thymic mTEC expression level versus average TCGA OV PANCAN expression. All mTEC-expressed genes are shown as black symbols. **(c)** HGSC shared auto-antibody target genes (red symbols) were examined for thymic mTEC expression level versus average TCGA OV HiSeq expression. All mTEC-expressed genes are shown as black symbols.

Supplementary Figure S2.

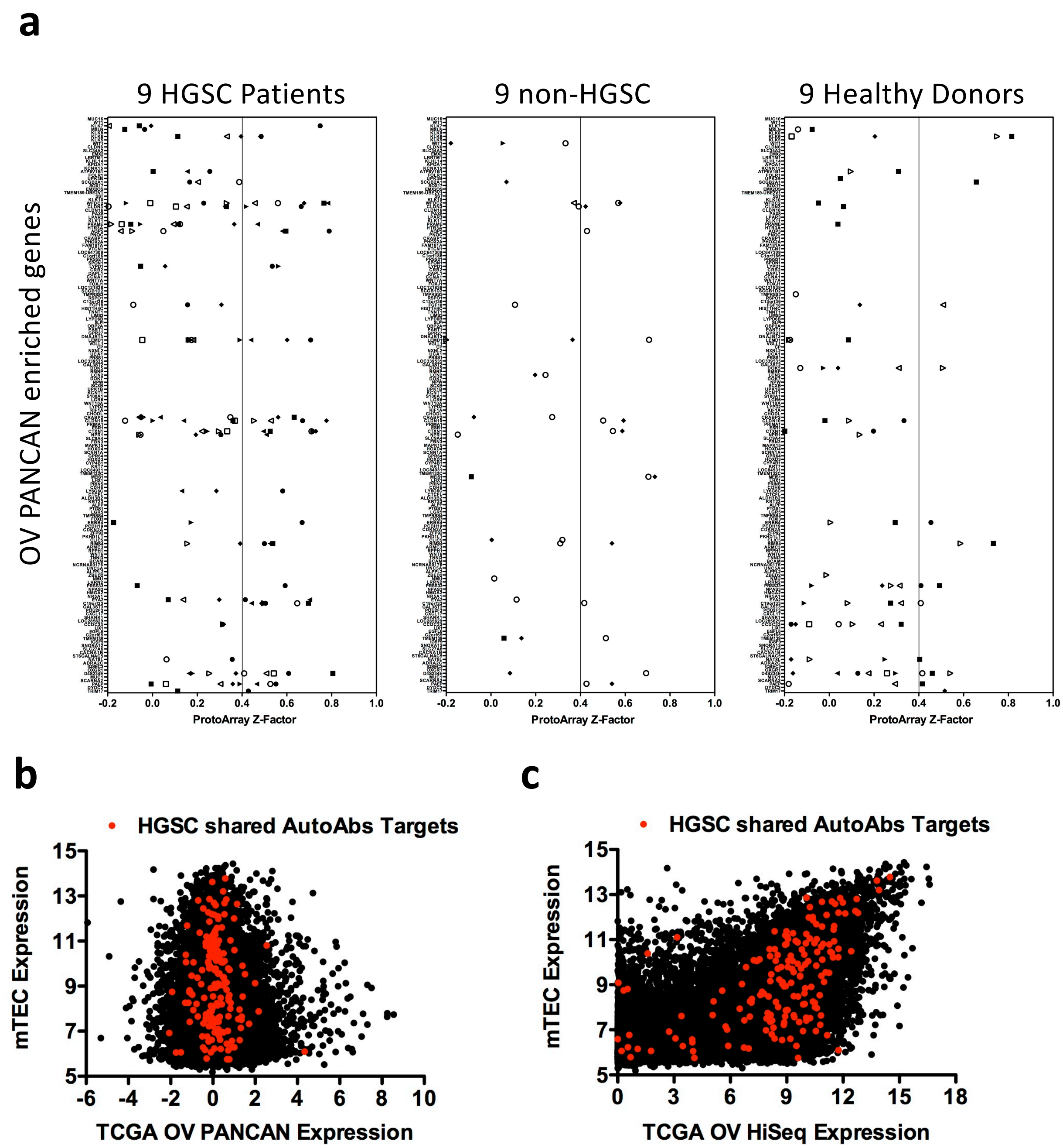

Supplementary Figure S3. Identification of Individual epitopes recognized by known HGSC patient TILs by custom peptide-MHC tetramer staining. **(a)** Following 14-day TIL expansion with TAA peptide pool, un-restimulated TILs were stained with individual tetramers and anti-CD8. Only patient TIL samples that recognized at least 1 peptide in the pool are shown. Each TAA peptide is indicated above the dot plots. The percentage of cells in each quadrant is indicated on each plot. Results are representative of 2-3 separate expansion and staining experiments. **(b)** Following 14-day TIL expansion with AutoAb-target peptide pool, un-restimulated TILs were stained with individual tetramers and anti-CD8. Only patient TIL samples that recognized at least 1 peptide in the pool are shown. Each AutoAb-target peptide is indicated above the dot plots. The percentage of cells in each quadrant is indicated on each plot. Results are representative of 2-3 separate expansion and staining experiments.

Supplementary Figure S3.

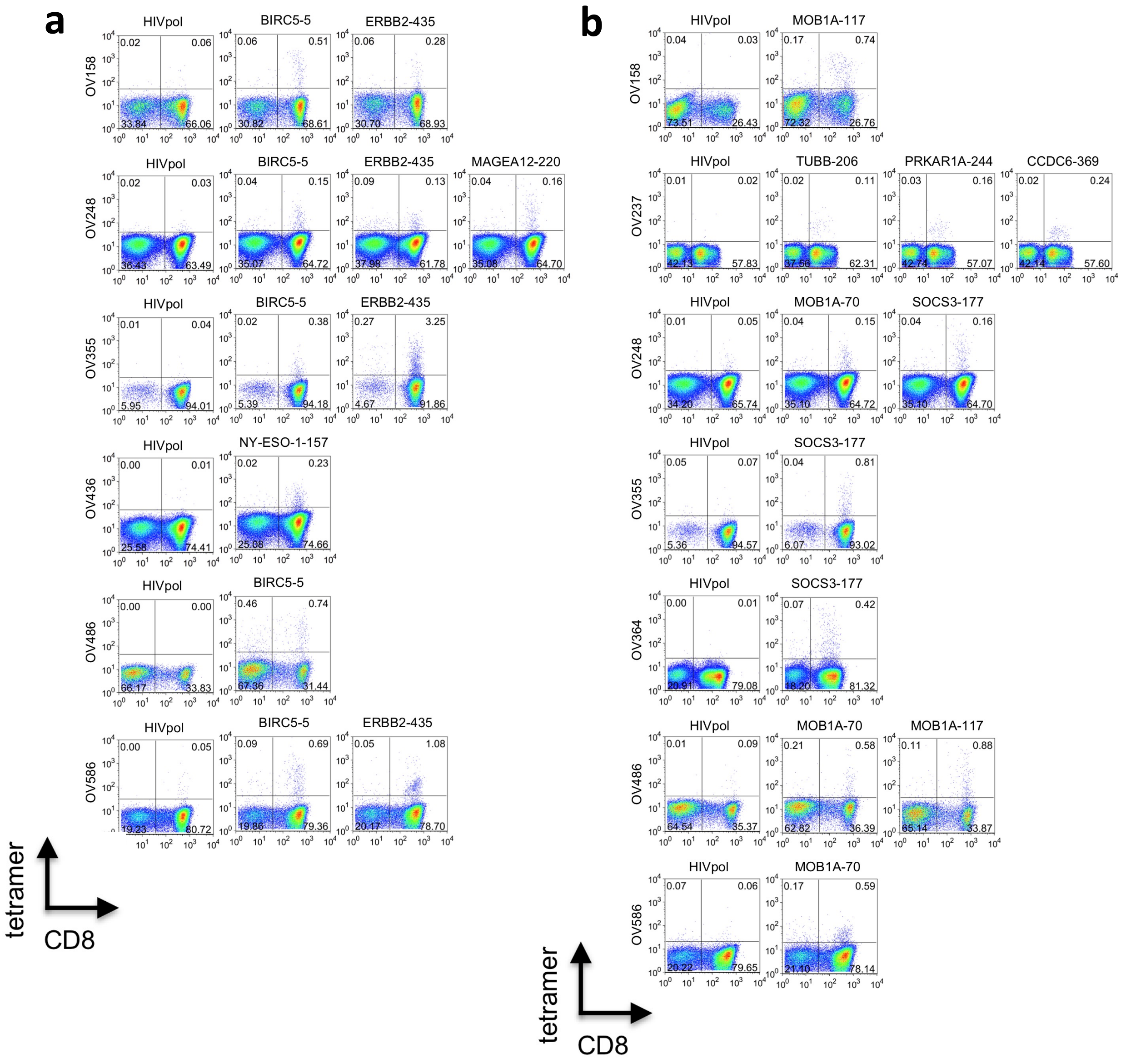

Supplementary Figure S4. Individual HGSC patient tumor and thymus expression level of somatic SNV mutated genes. **(a)** Thymic mTEC expression versus patient-specific gene-expression (RNA-Seq TPM) plots of the auto-antibody repertoire for each HGSC patients are shown, with individual tumor SNV mutated genes highlighted as red symbols. **(b)** Total somatic tumor SNV mutated genes from 9 HGSC patients (highlighted as red symbols) were examined against all mTEC expressed genes on thymic mTEC expression level versus TCGA OV PANCAN expression plots. **(c)** Total somatic tumor SNV mutated genes from 9 HGSC patients (highlighted as red symbols), were plotted against all thymic mTEC expressed gene expression (black symbol) versus average TCGA OV HiSeq expression.

Supplementary Figure S4.

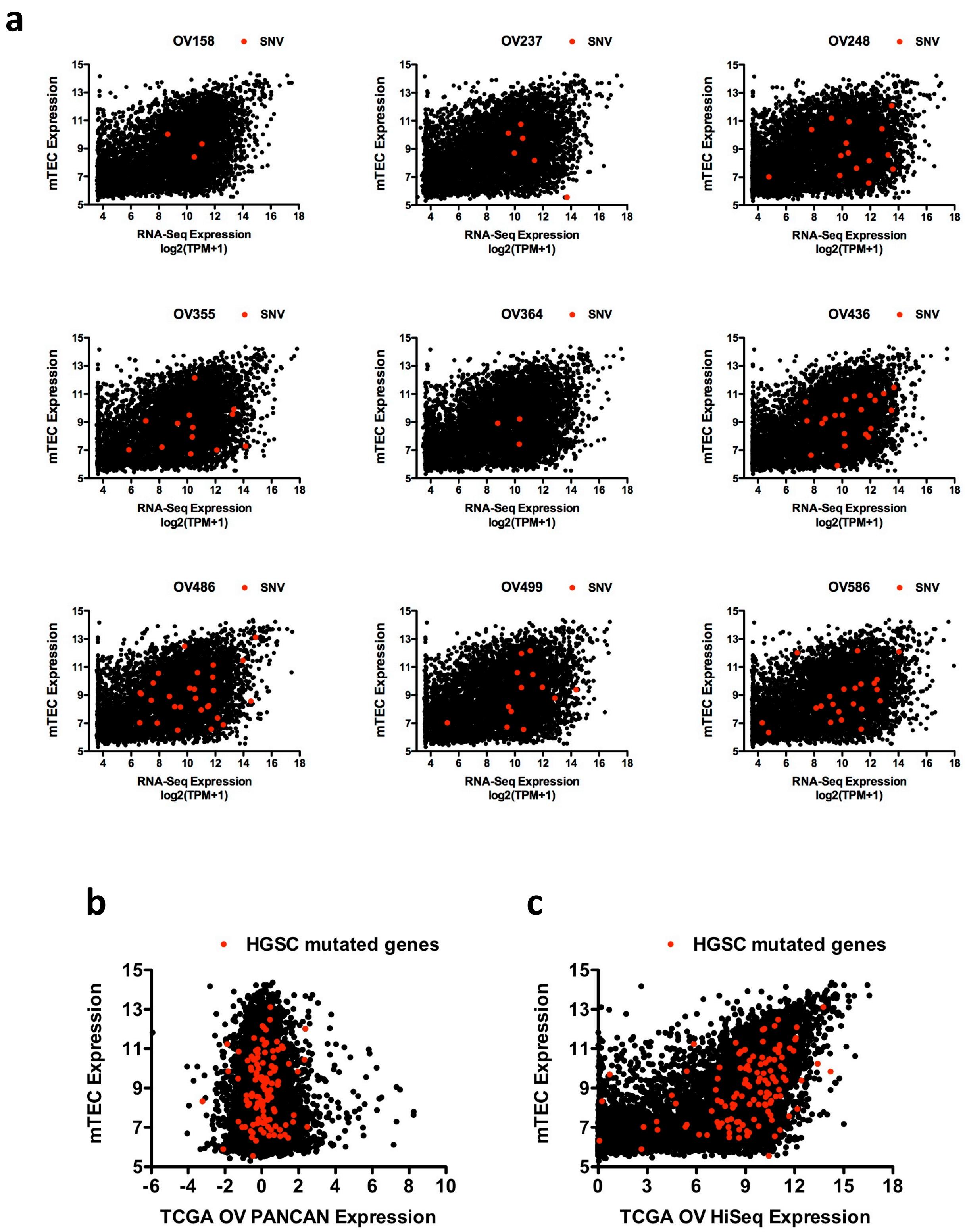

Supplementary Figure S5. Lack of detectable expansion of neo-epitope specific TILs. Following 14-day TIL expansion with predicted SNV peptide pools, the frequency of neo-epitope specific TILs was examined by flow cytometry using custom pMHC tetramers. Un-restimulated TILs were stained with individual tetramers and anti-CD8. HIVpol peptide tetramer was used as a negative control and Mart-1 A27L tetramer served as a positive control. Each mutated peptide is indicated above the dot plots. The percentage of cells in each quadrant is indicated on each plot. Results are representative of 2-3 separate expansion and staining experiments.

Supplementary Figure S5.

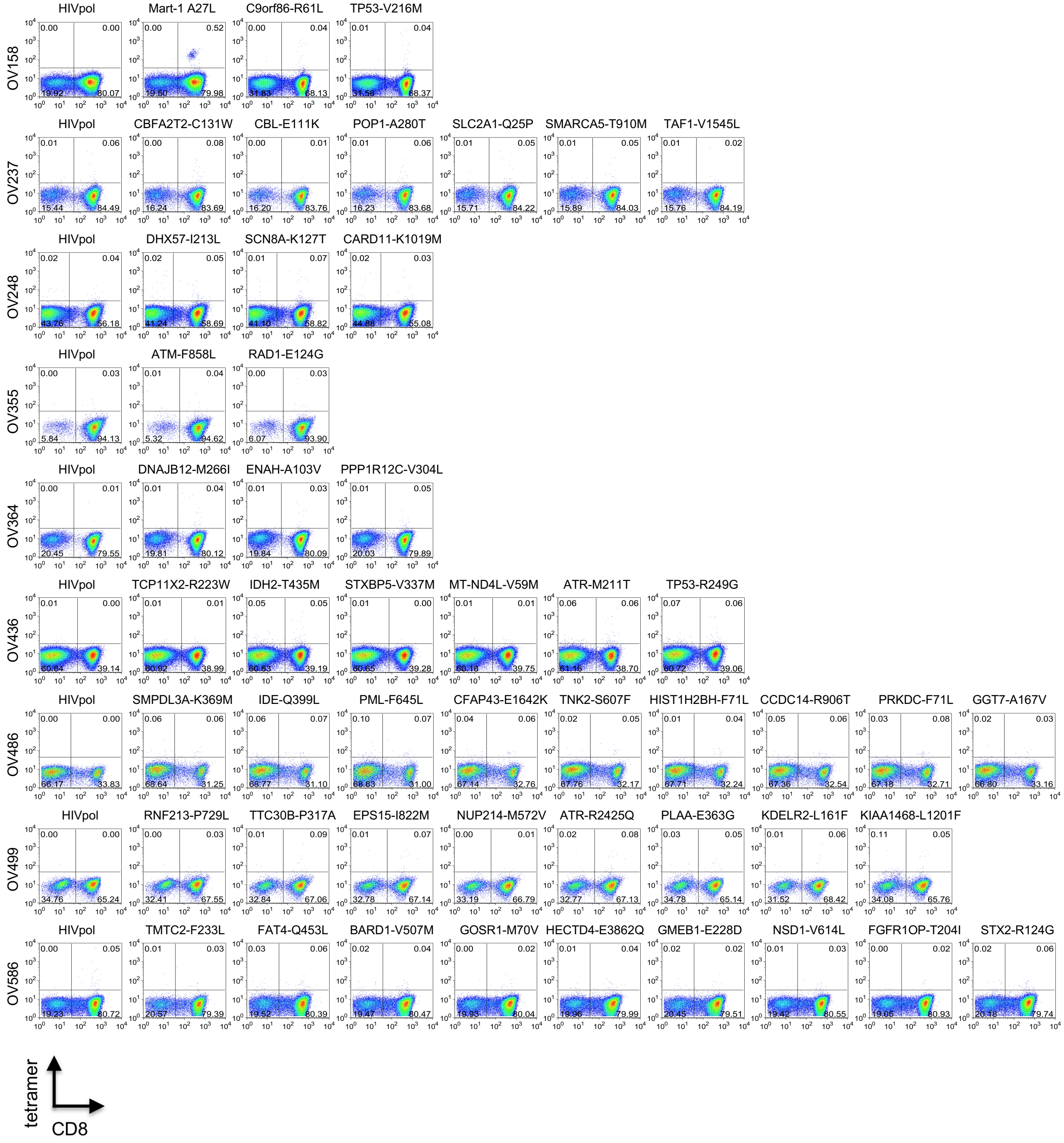

Supplementary Figure S6. High prevalence of pathogen-associated epitope recognition by HGSC patient TILs. Patient TILs were expanded for 14 days with A2+ donor DCs pulsed with the CEF pool of 7 pathogen associated epitopes from CMV, EBV, and Flu (see Supplementary Table S2A). Cultures were re-stimulated with DCs pulsed with individual peptides from the CEF pool for 5hrs in the presence of BFA. The re-stimulated cells were then stained for CD8 followed by intracellular staining for IFNγ production. Flow cytometry dot plots showing the percentage of CD8+ IFNγ+ cells are shown. Results are representative of 3 separate expansion and staining experiments.

Supplementary Figure S6.

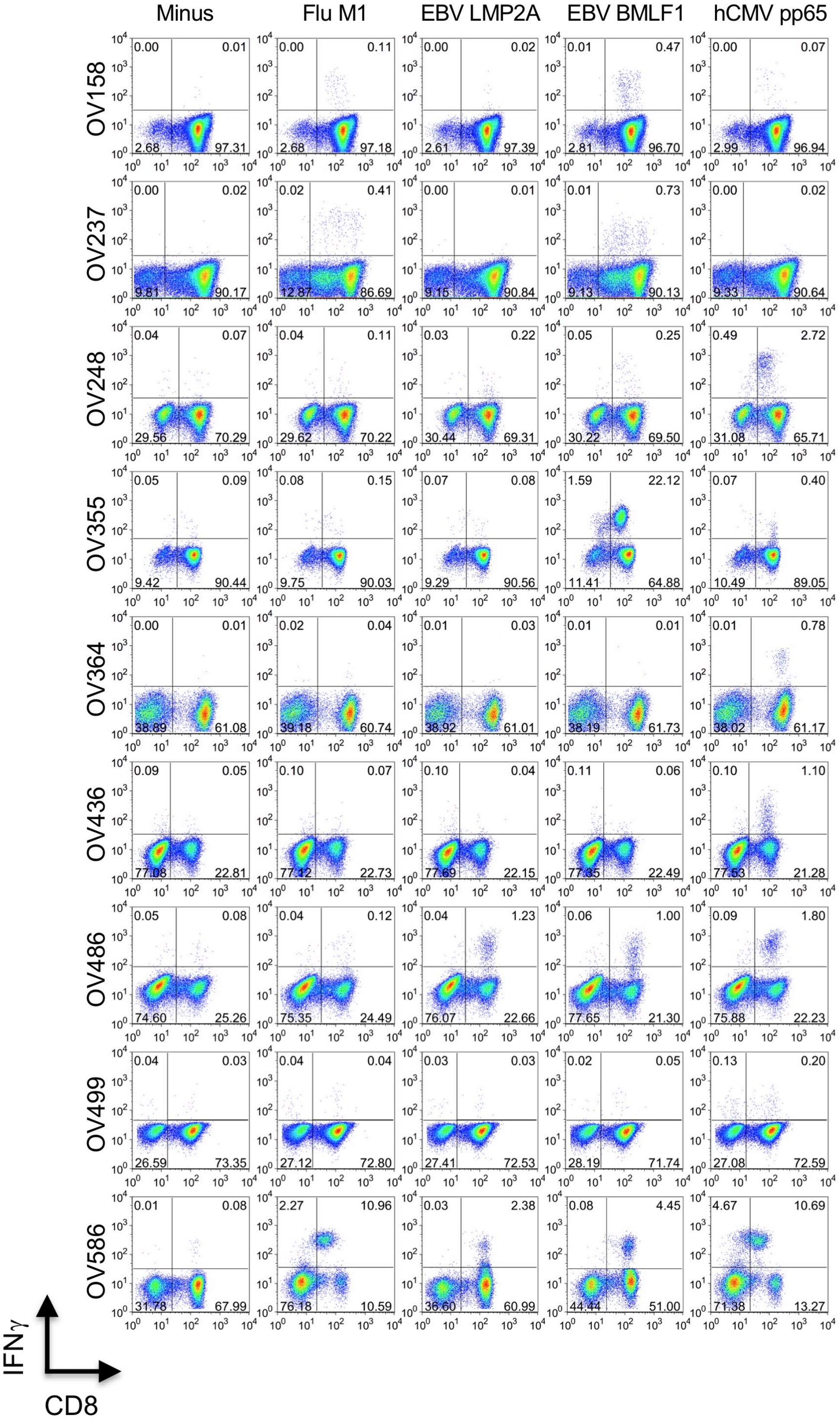

Supplementary Figure S7. Tumor and healthy tissue expression of novel candidate HGSC tumor associated antigens. Gene expression levels of **(a)** the 5 novel AutoAb-defined antigens; (**b**) the 4 known TAA; and **(c)** the additional 4 shared MAP-defined antigens identified in this study were examined across aggregated and normalized expression data from 33 cancer types with matched healthy tissues in TCGA and GTEx RNA-seq databases using the GEPIA web server (http://gepia.cancer-pku.cn). Tumor samples are shown in red, and normal samples are green.

Supplementary Figure S7.

a

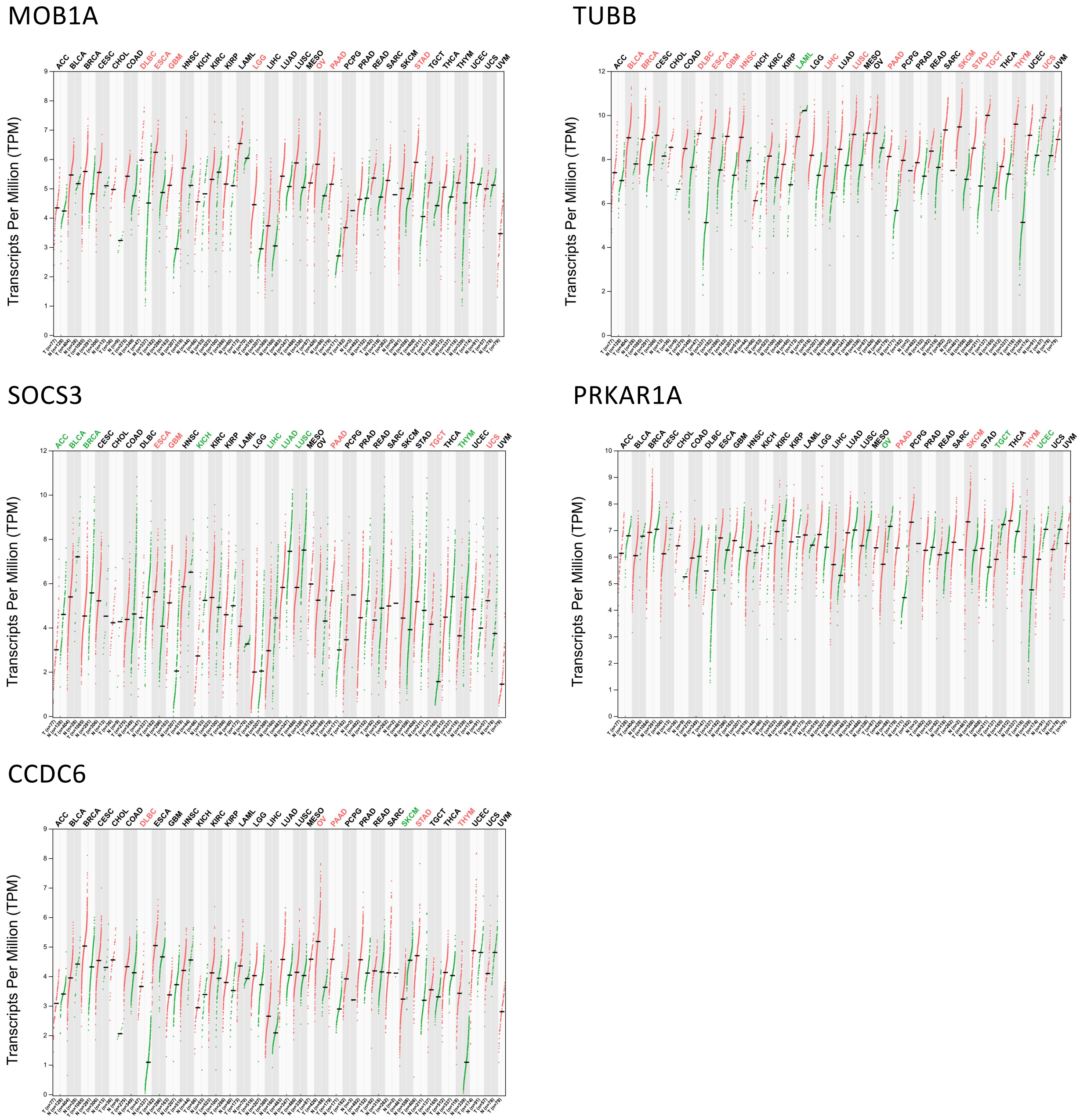

Supplementary Figure S7. continued

b

ABCF3

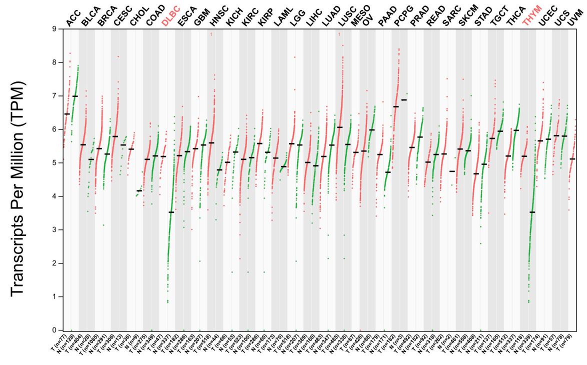

ANXA2

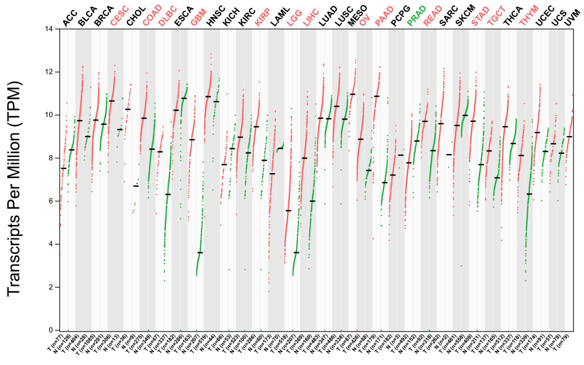

CRABP1

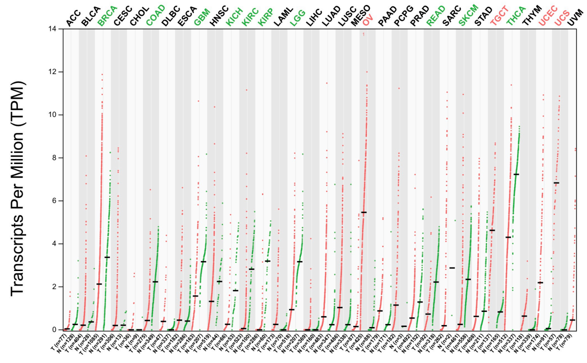

CRABP2

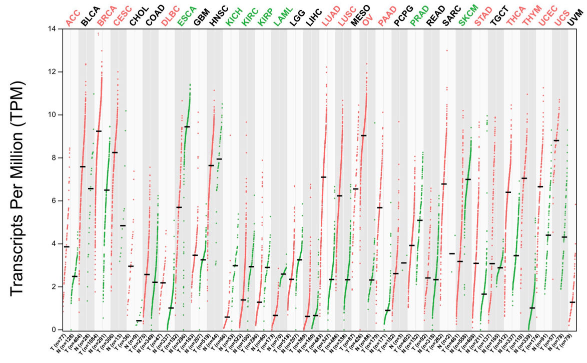

Supplementary Figure S7. continued

c

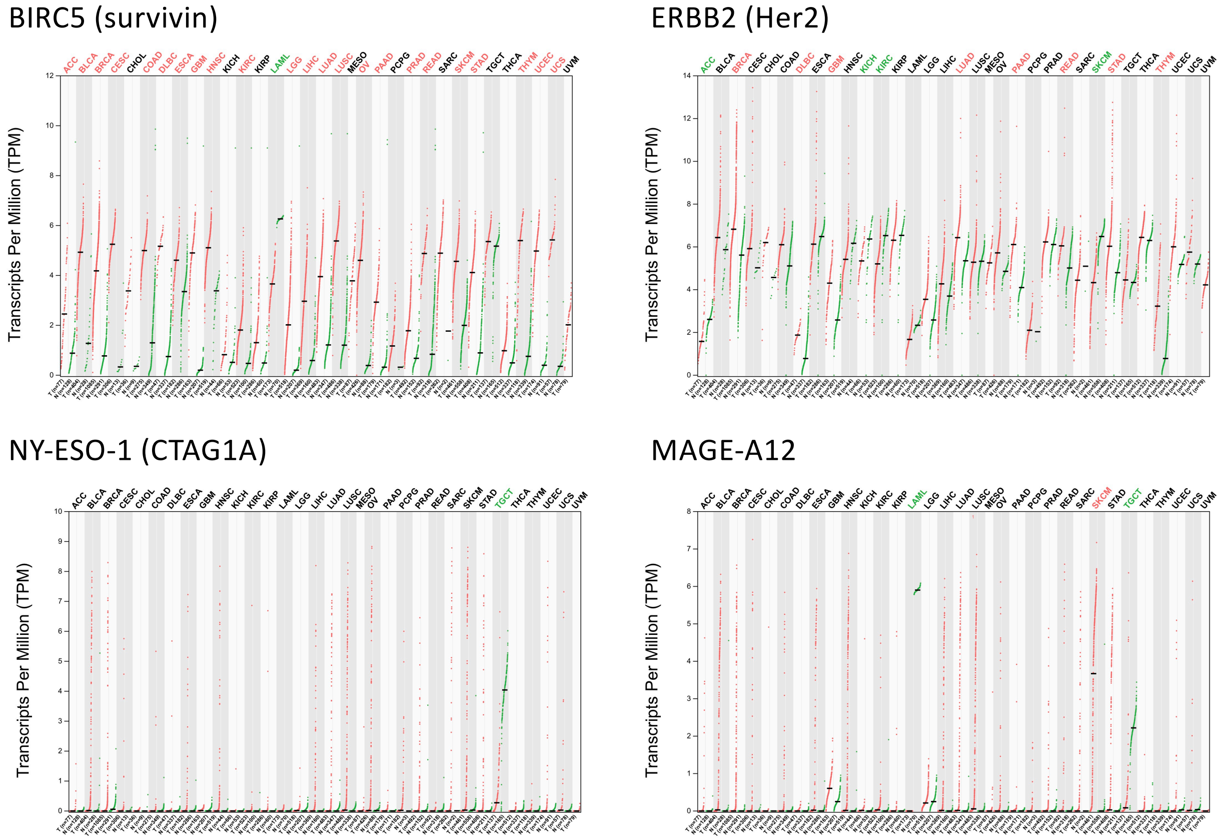

Supplement: Supplementary file 1 — Supplementary file1 (PDF 33581 KB) [file 262_2023_3413_MOESM1_ESM.pdf]
